# Modeling Reveals How Direct-Acting Antivirals Redirect HBV Capsid Assembly Pathways to Noninfectious Products

**DOI:** 10.64898/2026.05.25.727729

**Published:** 2026-05-26

**Authors:** Layne B. Frechette, Smriti Pradhan, Farzaneh Mohajerani, Carolina Pérez-Segura, Jodi A. Hadden-Perilla, Adam Zlotnick, Michael F. Hagan

**Affiliations:** 1 Martin Fisher School of Physics, Brandeis University, Waltham, Massachusetts 02453, USA; 2 Department of Chemistry & Biochemistry, University of Delaware, Newark, Delaware 19716, USA; 3 Molecular and Cellular Biochemistry Department, Indiana University, Bloomington, Indiana 47405, USA

## Abstract

Hepatitis B virus (HBV) infections cause chronic liver disease, resulting in about one million deaths per year, and there is currently no cure. Recent work has shown that a class of small molecules called capsid assembly modulators (CAMs) is promising for treating HBV. CAMs bind to HBV capsid protein subunits and alter their assembly, leading to non-functional and malformed structures rather than functional, closed shells. However, the mechanisms by which CAMs alter capsid assembly pathways remain unclear. Here, we extend a recently-developed kinetic Monte Carlo (KMC) model for HBV capsid assembly to simulate how CAMs affect assembly. In the model, CAMs alter assembly by preferentially binding to interfaces between certain quasi-equivalent subunit conformations. Simulations of the model reproduce experimental assembly product distributions. By analyzing assembly trajectories, we clarify the roles of thermodynamics and kinetics in determining assembly products, identify assembly mechanisms, and predict the key intermediates that lead to either capsids or malformed structures. Our findings enhance our fundamental understanding of capsid assembly, help advance the development of CAMs as a treatment for HBV and, more broadly, inform efforts to direct self-assembly pathways toward specific products.

## I. INTRODUCTION

During the lifecycle of many viruses, protein subunits self-assemble into closed shells (capsids) that enclose the viral genome and other viral components. Many viruses require precisely-controlled shapes and sizes to be infectious. For example, about half of all viruses have icosahedral capsids [1]. Yet, many viruses also exhibit polymorphism, or a tendency to form icosahedral capsids with different sizes or alternative, non-icosahedral structures [2-11]. Experiments and simulations have shown that small molecule antivirals [12, 13], as well as changing solution conditions [12, 14-17] or co-assembly with a cargo [12, 14-19] can affect which of these structures assemble. Understanding the mechanisms that control such polymorphic assembly would advance our understanding of the fundamental principles of self-assembly, aid in the rational design of drugs that misdirect virus assembly, and guide the development of synthetic self-assembly systems with tunable morphologies.

Icosahedral virus capsids are geometrically characterized by their triangulation number T, which (typically) corresponds to the number of different ‘quasi-equivalent’ conformations needed to assemble the capsid [20-22]. A capsid with triangulation number T has 60T subunits, with 60 subunits in each of the T conformations.

Hepatitis B virus (HBV) is a particularly interesting and important example of viruses that exhibit polymorphism. HBV is a major cause of human disease. Although an effective vaccine exists, hundreds of millions of people still suffer from chronic HBV infections, and in 2022, 1.1 million people succumbed to complications of HBV, including cirrhosis and liver failure [23]. There is currently no cure for HBV, but a number of therapeutics are under development [24]. Among these, capsid assembly modulators (CAMs) — small molecules that bind to the capsid subunits and alter their assembly properties — are particularly promising [25, 26]. CAMs can be divided into two major classes: CAM-Es, which accelerate assembly to form capsids that are morphologically normal but contain no genetic material [27-29], and CAM-As, which both accelerate and misdirect assembly to yield ‘aberrant,’ malformed structures [30, 31]. In this paper, we focus on CAM-As (which we abbreviate to ‘CAMs’ throughout), although our modeling framework can easily incorporate CAM-Es. Of the various CAM-As, heteroaryldihydropyrimidines (HAPs) have especially striking effects on HBV assembly behavior [12, 30]. HBV capsids are naturally dimorphic — while the majority of capsids that form in host cells have T=4 structures, approximately 5% have T=3 structures [32-34]. In vitro assembly of capsids leads to similar polymorphism, although the relative abundances of these polymorphs depends on the salt concentration, with higher salt concentrations correlating with progressively stronger association energy [14, 15, 35]. Kondylis et al. [12] showed that HAP-TAMRA (a fluorophore-labeled HAP [36] that serves as a model to study broader HAP-type CAM-A behavior) abolishes T=3 capsid assembly and promotes the assembly of malformed structures, including large, extended sheets and structures containing a mix of capsid-like and sheet-like motifs. Investigating three regimens of low (80 mM), moderate (300 mM), and high (1000 mM) ionic strength, they also found that the relative abundances of T=4 capsid and malformed structures depend on the salt concentration. The factors that determine these assembly products, and the underlying assembly pathways, remain unclear.

Computational and theoretical models have yielded important insights into the dynamics and assembly of HBV capsids with and without CAMs. Atomistic simulations of whole assembled capsids have shown that HBV capsid subunits are remarkably flexible [37], and that bound CAMs can significantly distort the capsid geometry [38, 39]; a survey of experimental structures supports this prediction [40]. Coarse-grained simulations [35] enabled studying assembly pathways in the absence of CAMs, identifying long-lived intermediate structures and ‘hub states’ at which T=3 and T=4 assembly pathways diverge. Theoretical models [41, 42] have explored the roles of curvature free energy in polymorphism. Coarse-grained simulations [35] and theory [42] have helped explain how salt concentration affects the ratio of T=3 to T=4 capsids in the absence of CAMs.

A recent study by Kra et al. [13] combined time-resolved SAXS experiments, theory, and coarse-grained simulations to study how CAMs impact HBV assembly. The authors studied two different CAMs, JNJ-632 and Bay 41–4109: the latter induces assembly of large, non-T=4∕T=3 shells, and both accelerate capsid assembly. Their models of SAXS data suggested that CAM binding accelerates assembly by increasing the subunit-subunit binding free energy, consistent with previous experiments [31]. They also performed coarse-grained simulations in which an assembling capsid is represented as a growing elastic shell. By varying the shell’s radius of curvature and Föppl-von Kármán number (which sets the ratio of stretching to bending moduli), they identified regimes where the shell assembled into T=4 capsids, T=3 capsids, and elongated shapes resembling the structures seen in experiments with Bay 41–4109. Because the elongated shapes formed at high Föppl-von Kármán numbers, Kra et al. [13] proposed that Bay 41–4109 could induce aberrant structures by changing the elastic modulus of a growing capsid. However, since their simulations did not explicitly represent CAM binding, they could not investigate this proposal or other mechanisms by which CAMs alter assembly pathways.

In this work we use computational modeling to understand the effect of CAMs on assembly pathways and products. We extend a coarse-grained, kinetic Monte Carlo (KMC) model that some of us previously developed to study HBV capsid assembly in the absence of CAMs [35]. The model was parametrized using experimental and atomistic simulation data, enabling it to capture protein-protein binding affinities and the mechanical and dynamical properties of capsids. The simulations are highly computationally tractable, which enables harvesting many assembly trajectories and analyzing the distribution of assembly pathways and outcomes. In the absence of CAMs, the model correctly predicted HBV dimorphism, how the relative abundance of T=3 and T=4 depends on solution conditions, and mechanisms of error correction by which overgrown structures shed excess subunits and close their shells.

In the extended model, CAMs bind to the interface between HBV dimers with an affinity that depends on the capsid protein conformation, based on experimental data. The KMC simulations qualitatively reproduce experimental assembly size distributions in the presence and absence of CAMs across a range of ionic strengths. In particular, the results are consistent with the experimental observation that CAMs lead to aberrant capsid sizes at moderate salt conditions (300mM NaCl, moderate association energy), but that high salt (1000mM NaCl, strong association energy) ‘rescues’ assembly of native-sized capsids. Because our simulations resolve the assembly/disassembly of individual dimers and CAMs, our analysis reveals assembly mechanisms and how CAMs disrupt them. Our results suggest that CAMs misdirect assembly by stabilizing subunit conformations that are incompatible with closed T=4 or T=3 capsids, and instead promote the formation of large sheet-like structures, consistent with electron microscopy observations. Moreover, using Markov state model analysis, we identify key assembly intermediates, including ‘hub states’ at which pathways between different assembly products diverge. Importantly, these predictions can be tested by experiments. By revealing the mechanism of action of CAMs, the computational results provide critical information for improving existing CAMs or designing new ones.

## II. RESULTS

**FIG. 1.**
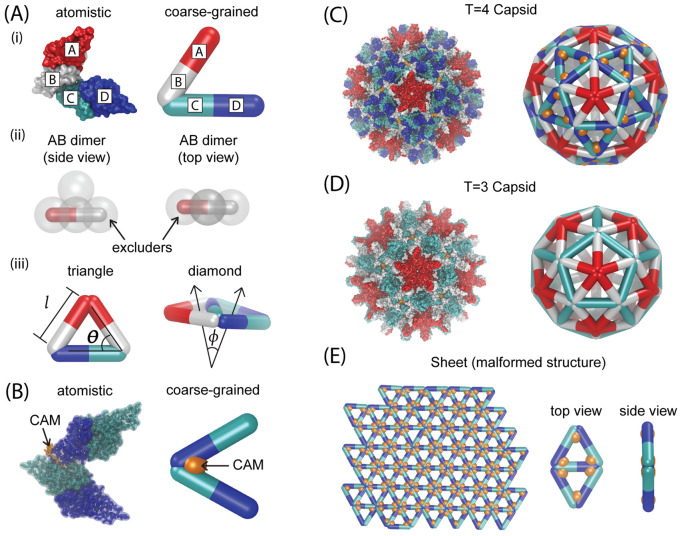
Overview of the coarse-grained model. (**A**) (i) Illustrations of atomic-resolution (left) and coarse-grained (right) dimer-of-dimers structures. The atomistic structure is extracted from the crystal structure of a complete capsid (PDB ID: 6BVF). Letters A, B, C, and D denote the four different quasi-equivalent conformations found in the T=4 capsid. Here, an AB dimer (A in red, B in white) is bound to a CD dimer (C in cyan, D in blue). In the coarse-grained model, dimers are represented as edges in a flexible triangular mesh. (ii) Side (left) and top (right) views of a coarse-grained AB dimer. Transparent spheres are coarse-grained “excluder” particles. The excluders (of which there are four per dimer) are arranged so as to capture the overall dimer shape, with three excluders equally spaced along an edge and one placed above the middle of the edge (representing the “spike” in the atomistic dimer structure). For clarity, excluder particles are omitted from all other snapshots of coarse-grained structures. (iii) Illustration of a coarse-grained trimer-of-dimers or “triangle” (left) and a pentamer of dimers or “diamond” (right). The edge length l, binding angle θ, and dihedral angle ϕ all fluctuate about rest values which depend on the conformations of the dimers involved. (**B**) Left: atomic-resolution structure of a dimer of CD dimers, with a HAP-TAMRA molecule (an example of a capsid assembly modulator (CAM)) bound to the inter-dimer interface (PDB ID: 6BVF). Right: Coarse-grained dimer of CD dimers with a CAM (orange sphere) bound to the inter-dimer interface. (**C**) Illustration of the T=4 capsid structure. Left: Atomistic structure of the T=4 capsid, with HAP-TAMRA (orange) bound to the B-C and C-D interfaces (PDB ID: 6BVF). Right: Coarse-grained structure of the T=4 capsid, with CAMs bound to the C-D interfaces. (**D**) Illustration of the T=3 capsid structure. Left: Atomistic structure of the T=3 capsid (PDB ID: 6BVN), with HAP-TAMRA (orange) bound to the B-C interfaces. Right: Coarse-grained structure of the T=3 capsid. (**E**) Illustration of sheet-like malformed structures. Left: Example of a sheet from a simulation of the coarse-grained model. Right: Top and side views of a CAM-bound diamond of CD dimers. The rest dihedral angle for this structure is zero.

We use a coarse-grained model to investigate the effects of CAMs on HBV capsid assembly. We have extended the elastic shell growth model developed in Ref. [35, 43] for HBV assembly in the absence of CAMs. Fig. 1 illustrates the model and compares the structures it forms to atomistic protein structures. Capsid protein dimers (Fig. 1Ai), the basic assembly subunits, are represented as edges in a flexible triangular mesh (Fig. 1Ai,ii,iii). CAMs are represented as beads that bind to dimer-dimer interfaces (Fig. 1B). We use a kinetic Monte Carlo (KMC) algorithm to simulate assembly and CAM binding/unbinding. The mesh assembles into T=4 capsids (Fig. 1C), T=3 capsids (Fig. 1D), or malformed structures such as sheets (Fig. 1E). The relative abundances of these different structures depends strongly on both solution conditions and the presence/absence of CAMs, as we will now describe. Details of the model and the KMC algorithm are given in Section IV.

### A. Simulations capture how CAMs and salt concentration affect assembly products

**FIG. 2.**
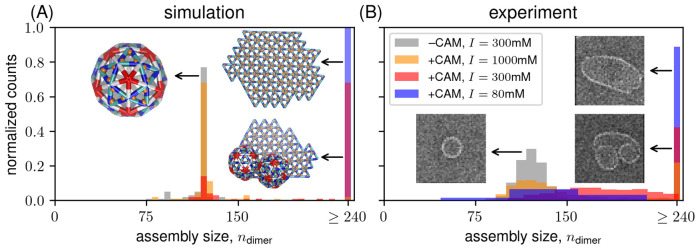
Simulations qualitatively reproduce experimental assembly product size distributions. Assembly product size distributions from (A) KMC simulations and (B) experiments [12] (data provided by S.C Jacobson). Results are shown for four different conditions: assembly without CAMs (−CAM) at an ionic strength of of I=300mM, and assembly with CAMs (+CAM) at three different ionic strengths (1000, 300, and 80mM). The plots show the number of assemblies of different sizes (measured in number of dimers per complex, $n_{\mathrm{dimer}}$), normalized by the total number of assemblies for each condition. Insets show representative structures (simulation snapshots in panel A and electron micrographs in panel B, adapted from Ref. [12], copyright 2018 ACS Publications) for the different peaks. Malformed structures typically include extended sheets dominated by a hexagonal repeating structure, often with partially formed, T=4–like shells at their periphery.

Fig. 2 compares the distributions of assembly product sizes from our KMC simulations to experimental size distributions from Kondylis et al. [12]. We show results with and without CAMs at an ionic strength of I=300mM, and with CAMs at higher (I=1000mM) and lower (I=80mM) salt concentrations. The simulations capture the key features observed in experiments: at I=300mM without CAMs, the predominant assembly products have $n_{\text{dimer}}\approx 120$, corresponding to T=4 capsids. The simulations also exhibit a small peak at ≈ 90 dimers, corresponding to a T=3 capsid; while T=3 capsids have been observed in experiments at these conditions [12], the resistive-pulse experiments used for Fig. 2B could not resolve T=3 from T=4 capsids. Adding CAMs dramatically alters the size distribution — most assemblies have $n_{\text{dimer}}>120$, and roughly half are nearly double that size ($n_{\text{dimer}}\geq 240$, shown as a single peak). These large sizes correspond to malformed structures which fail to form a closed capsid. Instead, they contain extended, sheet-like motifs, sometimes with partially-formed capsid-like structures present at the periphery of the sheets. Increasing the ionic strength to I=1000mM ‘rescues’ capsid assembly, partially restoring the peak at $n_{\text{dimer}}=120$, although a significant number of assemblies still have $n_{\text{dimer}}>120$. On the other hand, decreasing the ionic strength to I=80mM abolishes the $n_{\text{dimer}}=120$ peak and favors sheet-like assemblies, with most products having $n_{\text{dimer}}\geq 240$. Notably, all these trends are qualitatively consistent between the KMC and experimental results.

The simulations enable understanding the mechanisms underlying these experimental and computational observations. First, they can be partially understood from a thermodynamic perspective by considering how CAMs and salt concentration affect the free energies associated with dimer conformational changes and dimer-dimer binding. In our model, CAMs bind to C-D and D-C interfaces, stabilizing vertices with quasi-six-fold (hexameric) symmetry and hence favoring flat structures. This is consistent with the experimental size distributions in Fig. 2B and transmission electron micrographs showing flattening at quasi-six-fold axes in the presence of CAMs [12]. Increasing the salt concentration increases the total dimer-dimer binding affinity $g_{T=4}^{\text{bind}}$ (Table I). Based on previous experimental work [44] and comparison between previous KMC results without CAMs and experimental size distributions in Mohajerani et al. [35], the trend in binding affinity primarily reflects the fact that increasing salt concentration decreases the difference in conformational free energy between AB and CD dimers $\Delta g_{\text{CD}\to \text{AB}}^{\text{conf}}=-k_{B}T\;\log\;K_{\text{AB}}$, with $\Delta g_{\text{CD}\to \text{AB}}^{\text{conf}}$ decreasing by $2k_{B}T$ between I=80mM and I=1000mM (see Table I).

The thermodynamic factors discussed in the previous paragraph favor T=4 capsid formation at I=1000mM. However, our simulations show that thermodynamics alone cannot fully explain the rescue of capsids at I=1000mM, i.e. why T=4 capsids form instead of sheets. For example, if we artificially increase the CAM binding on- and off-rates while leaving the free energies unchanged with I=1000mM, we observe a significant proportion of malformed, sheet-like structures (see Fig. S1). This observation underscores the importance that CAM binding be slow in comparison to dimer-dimer binding kinetics to observe the ‘rescue’ of T=4 capsids under high salt, as previously suggested in Ref. [12].

Finally, we note that T=3 capsids are absent with CAMs at I=1000mM, although they make up a large fraction of assembly products *without* CAMs at I=1000mM in both experiments and simulations (see Ref. [12] and Fig. S2). Our model suggests a thermodynamic rationale for this observation: CAMs stabilize C-D and D-C interfaces, which are not present in T=3 capsids (see also Fig. 5 and the discussion of how CAMs destabilize T=3 intermediates in Section III, Subsection B.2).

The observation that the low salt concentration (I=80mM) favors sheets over T=4 capsids also involves a combination of thermodynamic and kinetic effects. The increasing conformational free energy cost $\Delta g_{\text{CD}\to \text{AB}}^{\text{conf}}$ makes the the AB conformation, and thus icosahedral capsids, less favorable (reduces the magnitude of $g_{T=4}^{\text{bind}}$, see Table I and SI Section S3), since the AB conformation is required for capsids but not sheets. From a kinetic perspective, destabilizing the AB dimers slows down T=4 assembly, even in the absence of CAMs (see Fig. S3). Then, adding CAMs not only thermodynamically stabilizes C-D and D-C interfaces, but also promotes rapid nucleation of sheet-like structures (compared to slow nucleation of T=4 capsids at low salt concentration without CAMs; see Fig. S3). Hence, sheets are also kinetically favored over T=4 capsids at I=80mM.

### B. CAMs alter assembly pathways

**FIG. 3.**
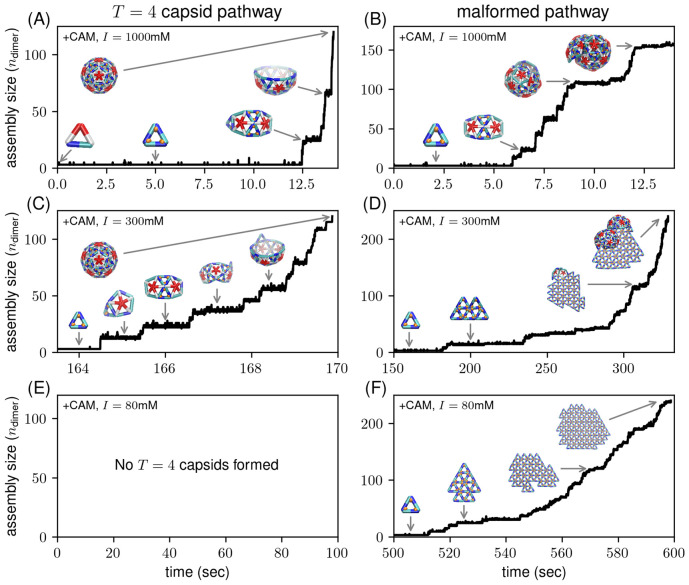
Changing salt concentration leads to different assembly pathways. The three rows show representative trajectories (assembly sizes versus time) leading to T=4 capsids (left column, panels A, C, E) and malformed structures (right column, panels B, D, F) at different salt concentrations. CAMs are present in all cases. Representative intermediate structures are shown as inset snapshots with arrows pointing to the corresponding time points on the trajectories. See Supplementary Movies S1-S5 for movies of the assembly trajectories shown in panels A, B, C, D, and F.

**FIG. 4.**
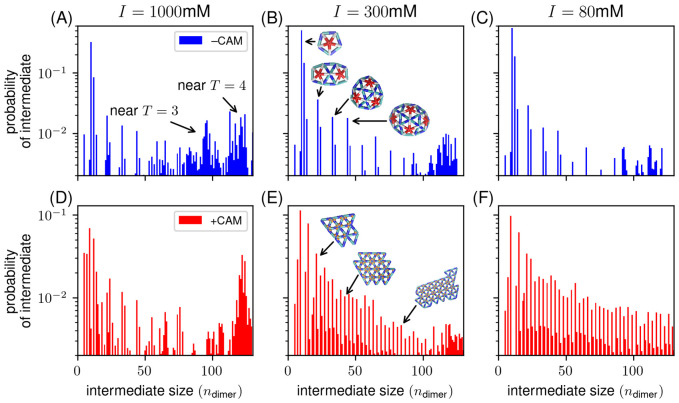
CAMs alter the prevalence of assembly intermediates. Probabilities of different assembly intermediates, normalized by the total number of intermediate structures (structures with size $n_{\mathrm{dimer}}>3$ which are neither T=3 nor T=4 capsids) over all trajectories at a given salt concentration and presence/absence of CAMs. The top row (panels A-C) shows results without CAM (−CAM, blue); the bottom row (panels D-F) shows results with CAM (+CAM, red). The three columns show results at different salt concentrations. Peaks corresponding to near-T=3 and near-T=4 capsid intermediates are labeled in panel A. In the middle column (panels B and E), snapshots illustrate representative structures associated with selected peaks in the distributions.

**FIG. 5.**
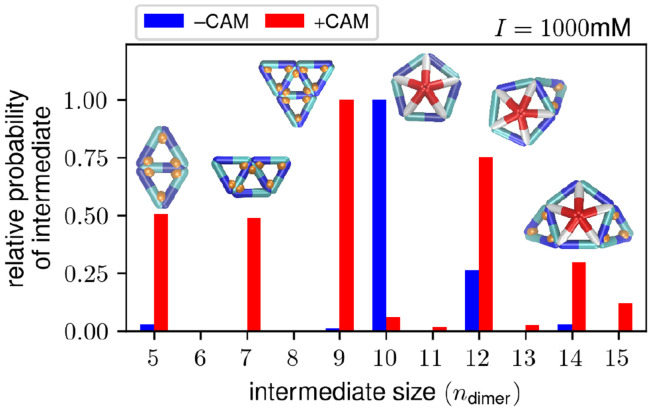
CAMs abolish T=3 capsid formation by altering the distribution of early assembly intermediate sizes. Probability of different assembly intermediates ($n_{\mathrm{dimer}}>3$, excluding T=4 or T=3 capsids), relative to the probability of the most probable intermediate, as a function of intermediate size (measured in number of dimers, $n_{\mathrm{dimer}}$). We compute probabilities by counting the number of assemblies of each size within all trajectories at a given set of conditions. We show results for simulations at I=1000mM without (blue) and with (red) CAMs. Snapshots illustrate representative intermediates corresponding to each peak.

Next, to mechanistically understand the effect of CAMs on assembly products, we investigate how CAMs change assembly pathways. Fig. 3 shows representative trajectories for different salt concentrations leading to either T=4 capsids or malformed structures (including sheets). We also provide movies of the assembly trajectories corresponding to these plots (Supplementary Movies S1-S5), see SI Section S5 for descriptions. Each panel in Fig. 3 shows the size (in number of dimers, $n_{\text{dimer}}$) as a function of time for a single trajectory. In all cases, assembly is preceded by a long nucleation process in which the size fluctuates about the initial $n_{\text{dimer}}=3$ nucleus. We show this phase explicitly in the top row (I=1000mM); in the bottom two rows this waiting period is typically much longer, and so we start the plots at times near the beginning of the growth phase. We see that trajectories leading to T=4 capsids proceed via a series of transiently stable intermediates which contain successively larger numbers of five-fold vertices (see Supplementary Movie S1). The same mechanism leads to T=4 capsids at I=1000 and 300mM (see Supplementary Movies S1 and S3), while no T=4 capsids assembled for I=80mM. By comparison to trajectories without CAMs (Ref. [35], Fig. S4, and Supplementary Movie S6), we see that the primary effect of CAMs on the T=4 trajectories is to stabilize C-D interfaces (and hence CD conformations) in the three-dimer nucleus and subsequent small oligomers. This alters the structure of the first stable intermediate with $n_{\text{dimer}}>3$: rather than the 10-dimer intermediate (which we henceforth refer to as the ‘decamer’) commonly observed in CAM-free capsid assembly (see Fig. 4B, Fig. 5, and Ref. [45]), we predominantly observe a 12-dimer intermediate (which we henceforth refer to as the dodecamer, see e.g. Fig. 5) in trajectories with CAMs. The dodecamer consists of a five-fold vertex with a CAM-bound dimer-of-CD-dimers. This structure is incompatible with a T=3 capsid (in which adjacent triangles associated with five-fold vertices share a CC dimer edge). Thus, CAMs disfavor T=3 capsid assembly by destabilizing the decamer, which is compatible with both T=3 and T=4 capsids, in favor of the dodecamer, which is compatible only with T=4 capsids (see Section II, Subsection B.1 for further discussion).

In contrast to T=4 capsids, both the final structures and pathways associated with malformed assemblies change with salt concentration. At I=1000mM, the predominant malformed structures are similar in size to T=4 capsids, but fail to close. This is usually due to mixed T=3∕T=4 morphologies (e.g. the final structure illustrated in Fig. 3B; see Supplementary Movie S2), which contain differing local curvatures that are incompatible with a closed shell. This class of pathways is very similar to the mixed-morphology malformed pathways we previously observed for assembly without CAMs [35]. However, we also observe other classes of malformed structures, including ‘dumbells’ consisting of two bound, partially-formed T=4 capsids, as well as mixed T=4/small sheet morphologies (see SI Fig. S5). At I=300mM, most malformed structures consist of extended sheets, often decorated at their edges with partial, T=4-like shells (final structure in Fig. 3D and Supplementary Movie S4). This suggests that the driving forces for sheet and T=4 formation are comparable in magnitude under these conditions. In contrast, for I=80mM, sheets become significantly more favorable (see Supplementary Movie S5). We rarely observe T=4-like partial shells, and sheet growth is generally faster than at I=300mM. While small ($n_{\text{dimer}}≲25$) sheet-like intermediates grow via distinct jumps between transiently stable intermediates, larger assemblies grow more steadily. This is because the number of potential sites for adding additional CD dimers grows with the perimeter of the sheet: since the sheet is flat (rather than curved, as for T=4 capsids) the perimeter increases steadily with the size of the sheet. The large number of available binding sites suggests that multiple sheet-like intermediates could associate to form larger malformed structures [46, 47], although we neglect this possibility in our model.

#### 1. CAMs alter the prevalence of key assembly intermediates

To better understand how CAMs modify and misdirect assembly pathways, we compute the probabilities of observing different intermediate structures during assembly. We define an intermediate as any structure with $n_{\text{dimer}}>3$ which is not a complete capsid. Fig. 4 shows the probability distributions of intermediates as a function of size at different salt concentrations, both without (−CAM, blue, top row) and with (+CAM, red, bottom row) CAMs. Each distribution exhibits a series of peaks with a ‘multiplet’ structure, consisting of clusters of closely spaced peaks. For assembly without CAMs (top row), most multiplets are separated from their neighbors at a regular interval of ≈ 11 dimers. These correspond to intermediates with successively larger numbers of five-fold vertices. The multiplet structure arises due to a transient loss or gain of one or two dimers from the most probable structure in each multiplet. This is consistent with the step-like growth observed in the trajectories in Fig. 3. Dense clusters of peaks occur around $n_{\text{dimer}}=90$ and 120, corresponding to near-T=3 or near-T=4 structures, respectively, awaiting the addition or removal of a few dimers to form a complete shell.

With CAMs, at I=1000mM we observe a similar multiplet structure as in the trajectories without CAMs, although there are more numerous smaller peaks (likely due to the addition of dimers-of-CD-dimers, stabilized by CAMs, to structures with successively larger numbers of five-fold vertices), and the near-T=3 peak is markedly reduced (consistent with the absence of T=3 capsids in the I=1000mM, +CAM histogram in Fig. 2).

At lower salt concentrations with CAMs, the intermediate probability distributions change dramatically. The near-T=4 peak is markedly reduced at I=300mM, reflecting the low yields of T=4 capsids, and is entirely absent at I=80mM, reflecting a total lack of T=4 capsids. Moreover, the multiplet spacing changes from $n_{\text{dimer}}=11$ to $n_{\text{dimer}}=3$, corresponding to the addition of triangles of CD dimers rather than additional five-fold vertices. Fig. 4B,E show snapshots corresponding to the peaks at I=300mM with and without CAMs, illustrating the structures of these intermediates. The intermediate probability distributions thus reflect the pathways by which different structures assemble. Importantly, the size distributions of assembly intermediates can be measured experimentally (e.g. [45, 48-51]), enabling a direct test of our predictions.

#### 2. Intermediate distributions reveal the mechanism by which CAMs suppress T=3 capsid assembly

A closer inspection of small intermediate probabilities reveals the mechanism by which CAMs abolish T=3 assembly. Fig. 5 shows the probability distributions of intermediates up to size $n_{\text{dimer}}=15$ for assembly with and without CAMs at I=1000mM. To facilitate comparison, we show each distribution normalized by the probability of the most likely intermediate for that system (thus, e.g. the probabilities for assembly without CAMs are normalized by the probability of the decamer). We show snapshots illustrating the most common structure at each size above the corresponding bars in the plot. We see that without CAMs, the most probable intermediate is the decamer, followed by the dodecamer, both of which have a single five-fold vertex. By contrast, the most probable intermediate with CAMs is a 9-mer consisting entirely of CD dimers (smaller all-CD 5-mer and 7-mer intermediates also feature prominently). The next most common intermediate is the dodecamer, and a 14-mer containing a single five-fold vertex with two additional dimers-of-CD-dimers is also common. Importantly, the probability of observing the decamer in trajectories with CAMs is tiny compared to the probability of the dodecamer and of the 14-mer. This observation provides strong evidence for the mechanism of abolishing T=3 capsid assembly that was suggested by assembly trajectories (Fig. 3): the decamer is compatible with both T=3 and T=4 capsids, but due to their CD dimers, the dodecamer and the 14-mer are compatible only with a T=4 capsid. Thus, by stabilizing the dodecamer and the 14-mer, CAMs greatly reduce the probability of forming a T=3 capsid (or any other 90-mers, see Fig. 4A,D).

#### 3. Intermediate distributions resolved by pathway identify small intermediates unique to different assembly pathways

**FIG. 6.**
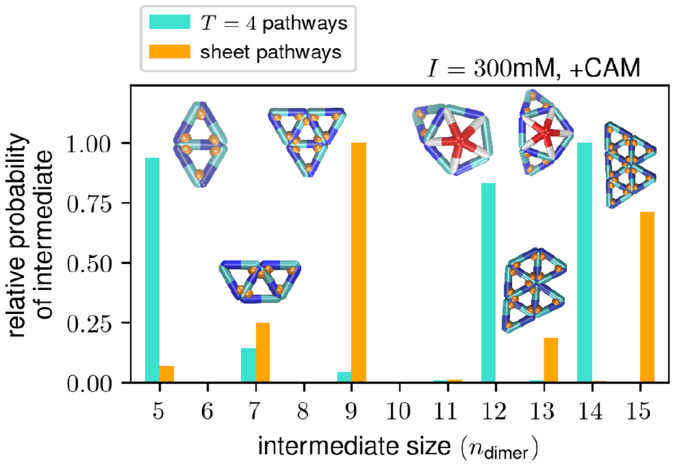
Small intermediates determine assembly products. Probability of different assembly intermediates ($n_{\mathrm{dimer}}>3$, excluding T=4 or T=3 capsids), relative to the probability of the most probable intermediate, as a function of intermediate size (measured in number of dimers, $n_{\mathrm{dimer}}$), for assembly with CAMs and I=300mM. We compute probabilities by counting the number of assemblies of each size within all trajectories with CAMs at I=300mM. The plot shows intermediate probabilities for pathways that end in T=4 capsids (turquoise) and sheets (orange). The insets show representative snapshots of intermediates corresponding to each peak.

The distributions in Fig. 4 illustrate the mechanism by which T=4 capsids and sheets grow, but at what point does an assembly ‘choose’ one outcome over the other? This question is particularly relevant to assembly with CAMs at I=300mM, where both T=4 capsids and sheets form in significant numbers. To answer it, we partition trajectories into those that end in T=4 capsids and those that end in sheet-like structures. We define a sheet-like structure as any structure which at least 10 ‘sheet-like CD dimers,’ in which the CD dimer edge is shared by two triangles, both of which consist solely of CD dimers (see e.g. Fig. 1E). (By contrast, ‘T=4-like CD dimers’ form an edge that is shared by a triangle consisting of only CD dimers and a triangle with two AB dimers and one CD dimer; see Fig. 1Aiii, right.) This definition accounts for the fact that many malformed structures at I=300mM contain both T=4-like and sheet-like regions. We then compute intermediate probability distributions separately for trajectories ending in T=4 capsids and trajectories ending in sheet-like structures (Fig. 6). T=4 trajectories (turquoise) prominently feature the dodecamer and 14-mer, as well as a sheet-like 5-mer. In contrast, sheet-like pathways (orange) prominently feature the 9-mer, as well as the 7-mer and the larger 13-mer and 15-mer. Importantly, while the distributions exhibit some overlap for $n_{\text{dimer}}\leq 9$, there is no overlap for $n_{\text{dimer}}\geq 12$. Thus, sheet-like 13-mers almost never occur in T=4 pathways, while dodecamers (with their five-fold vertices) almost never occur in sheet pathways. This suggests that the dodecamer is ‘committed’ to forming (i.e. most probably going to form) a T=4 capsid (or malformed capsid) rather than a sheet, while the 13-mer is committed to forming sheet-like structures. Thus, assembly outcomes appear to be determined by which small intermediate – a T=4-like dodecamer or a sheet-like 13-mer – forms first.

#### 4. Committor probabilities identify small intermediate ‘hub states’ that determine assembly outcomes

To test whether different intermediates are indeed committed to forming certain structures, we computed committors for different intermediates. For a dynamical process involving transitions between a reactant state A and product state B, the committor probability, $q_{i}^{B}$, is the probability that a trajectory initiated in a state i reaches state B before state A [52, 53]. To compute committor probabilities, we first constructed a Markov state model (MSM) for HBV assembly with CAMs (see Section IV F for full details). An MSM coarse-grains a system’s dynamics into Markovian transitions between discrete microstates. We used a physically transparent discretization in which microstates $i\;=\;(n_{\text{AB}},n_{\text{CD}}^{T=4},n_{\text{CD}}^{\text{sheet}})$ are characterized by the number of AB dimers ($n_{\text{AB}}$), the number of T=4-like CD dimers ($n_{\text{CD}}^{T=4}$), and the number of sheet-like CD dimers ($n_{\text{CD}}^{\text{sheet}}$) in an assembly. We used the transition matrix T(τ) obtained from the MSM to compute committor probabilities $q_{i}^{T=4}$, $g_{i}^{\text{sheet}}$ associated with reaching the product states (T=4 capsid and sheet, respectively) before either returning to the reactant state (the initial, three-dimer nucleus) or ending in an alternative product state (e.g. a malformed capsid) (see Section IV G for details).

**FIG. 7.**
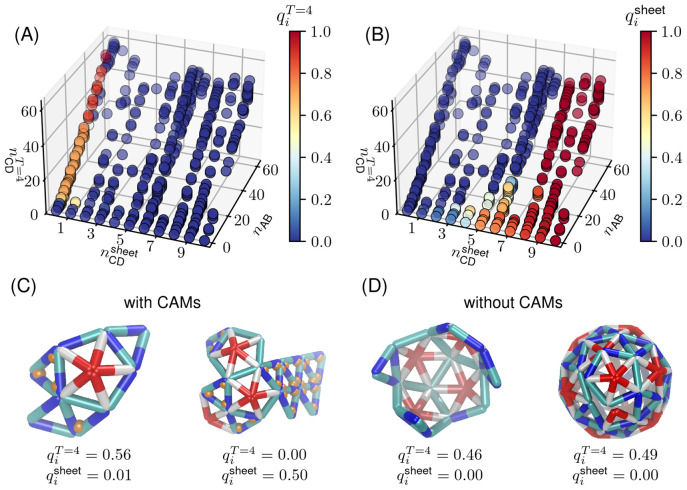
Committor probabilities enable identifying hub states for T=4 and sheet assembly. (A) Committor probabilities $q_{i}^{T=4}$ for T=4 capsid assembly as a function of $n_{\mathrm{AB}}$, $n_{\mathrm{CD}}^{T=4}$, and $n_{\mathrm{CD}}^{\text{sheet}}$. The color indicates the value of $q_{i}^{T=4}$. (B) Committor probabilities $q_{i}^{\text{sheet}}$ for sheet assembly (any structure with $n_{\mathrm{CD}}^{\text{sheet}}\geq 10$) as a function of $n_{\mathrm{AB}}$, $n_{\mathrm{CD}}^{T=4}$, and $n_{\mathrm{CD}}^{\text{sheet}}$. (C) Representative snapshots of T=4 capsid hub states with $q_{i}^{T=4}\approx 0.5$, $q_{i}^{\text{sheet}}\approx 0$ (left, $n_{\mathrm{AB}}=5$, $n_{\mathrm{CD}}^{T=4}=3$, $n_{\mathrm{CD}}^{\text{sheet}}=1$) and sheet hub states with $q_{i}^{\text{sheet}}\approx 0.5$, $q_{i}^{T=4}\approx 0$ (right, $n_{\mathrm{AB}}=13$, $n_{\mathrm{CD}}^{T=4}=6$, $n_{\mathrm{CD}}^{\text{sheet}}=6$). The committor probabilities are listed below the snapshots. In panels (A-C), there are CAMs and I=300mM. (D) Representative snapshots of T=4 capsid hub states without CAMs at I=300mM. Both the left and right snapshots have $q_{i}^{T=4}\approx 0.5$, $q_{i}^{\text{sheet}}\approx 0$ (sheets do not form without CAMs).

Plots of $q_{i}^{T=4}$ and $q_{i}^{\text{sheet}}$ as a function of $n_{\text{AB}}$, $n_{\text{CD}}^{T=4}$, and $n_{\text{CD}}^{\text{sheet}}$ are shown in Fig. 7A,B. When $n_{\text{CD}}^{\text{sheet}}=0$ and $n_{\text{AB}}\geq 5$, $q_{i}^{T=4}$ increases steadily with $n_{\text{AB}}$ and $n_{\text{CD}}^{T=4}$. Note that while for most near-T=4 states (with $n_{\text{AB}}$ and $n_{\text{CD}}^{T=4}$ both close to 60) $q_{i}^{T=4}\approx 1$, there are some states with $q_{i}^{T=4}\approx 0$, corresponding to malformed capsids. For nearly all microstates with $n_{\text{CD}}^{\text{sheet}}>0$, $q_{i}^{T=4}\approx 0$. Conversely, $q_{i}^{\text{sheet}}\approx 0$ for $n_{\text{CD}}^{\text{sheet}}=0$, but steadily increases with increasing $n_{\text{CD}}^{\text{sheet}}$. In particular, for the dodecamer, $q_{i}^{T=4}\approx 0.64$ and $q_{i}^{\text{sheet}}\approx 0.01$; for the 13-mer, $q_{i}^{T=4}\approx 0$ and $q_{i}^{\text{sheet}}\approx 0.74$. This confirms that the five-fold-vertex-containing dodecamer is indeed committed to forming a T=4 capsid, while the sheet-like 13-mer is committed to forming a sheet. Additionally, we identify “hub states” as intermediates for which either $q_{i}^{T=4}\approx 0.5$ or $q_{i}^{\text{sheet}}\approx 0.5$, i.e., they are equally likely to either form the structure of interest (T=4 capsid or sheet) or not. We show snapshots of representative hub states in Fig. 7C. The left state has $q_{i}^{T=4}\approx 0.5$, $q_{i}^{\text{sheet}}\approx 0$, contains a single five-fold vertex, and has $n_{\text{CD}}^{\text{sheet}}=1$. For the right state, $q_{i}^{T=4}\approx 0$, $q_{i}^{\text{sheet}}\approx 0.5$; the structure has two five-fold vertices and a small sheet-like domain. Interestingly, there are no hub states with both $q_{i}^{T=4}\approx 0.5$ and $q_{i}^{\text{sheet}}\approx 0.5$, suggesting that trajectories must return to the initial three-dimer nucleus to switch between T=4 and sheet assembly pathways. In Fig 7D we show representative hub states for assembly without CAMs, obtained by constructing an MSM from trajectories without CAMs at I=300mM and identifying states with $q_{i}^{T=4}\approx 0.5$ (see Fig. S6 for committor plots). In contrast to the hub states with CAMs shown in Fig. 7C, the hub states without CAMs exhibit mixed T=4∕T=3 morphologies, consistent with the results of Mohajerani et al. [35]. Thus, without CAMs, assembly outcomes are determined by a competition between locally T=3-like and T=4-like morphologies, whereas with CAMs they are instead shaped by a competition between locally sheet-like and T=4-like morphologies.

## III. DISCUSSION

We have presented a computational framework that, while parameterized from atomistic simulations, is sufficiently tractable to simulate assembly timescales. We have shown that the model predicts assembly kinetics and products consistent with experiments on the effect of small molecule antiviral agents (CAMs) on HBV assembly. The model results enable explaining how assembly products depend on salt concentrations and CAMs in terms of the thermodynamic affinities and kinetic binding rates of capsid protein dimers and CAMs, as well as the effects of CAM binding on the local geometry of a growing partial capsid. Moreover, the model makes experimentally testable predictions about how CAMs affect assembly pathways as a function of salt concentration, including the structures and concentrations of specific intermediates.

In this section, we further discuss how the model predictions can be experimentally tested, limitations of the current model and how they might be overcome, and potential applications to other assembly systems.

### A. Testing in experiments

While the predicted assembly product distributions are consistent with existing experiments (Fig. 2), our simulations also provide extensive predictions that could be tested by further experiments. These include assembly kinetics and predictions of assembly intermediate populations and lifetimes, which could be tested using high-resolution, time-resolved experimental techniques, such as time-resolved SAXS [45], resistive pulse sensing [48-51], interferometric scattering [54-57], and mass photometry [58-60]. Consistency of these experimental measurements with our predictions would support our proposed mechanisms, including how CAMs suppress T=3 capsid formation, and which intermediates are hub states, e.g. lead to T=4 capsids versus sheets.

### B. Limitations of the model

We have made the simplifying assumptions that CAMs only bind to interfaces between CD dimers, and that the only effect of CAM binding is to increase the net dimer-dimer binding affinity. However, experimental evidence and all-atom simulations show that some CAMs (including HAPs) not only increase dimer-dimer affinities, but also distort dimer-dimer binding angles, favoring the flat angles found at quasi-sixfold axes. For example, HAP-TAMRA and HAP18 asymmetrically distort capsids, flattening CD conformations and leading to faceted structures [36, 39]. The binding of HAP-ALEX (another fluorophore-labeled HAP) to intact T=4 capsids exhibits negative cooperativity [61], suggesting that distortions in binding geometries are correlated beyond neighboring subunits. Our model partially accounts for this effect by having CAMs bind only to the CD dimer conformations present at the quasi-sixfold axes. The fact that $g_{D\text{-}C}$ needs to be carefully tuned within a range $-4.88k_{B}T$ to $-4.93k_{B}T$ — small enough to destabilize T=4 capsids under normal assembly conditions (without CAMs), but large enough to allow sheets to grow when CAMs are present, suggests that this parameter may be qualitatively accounting for more complex conformational changes during assembly.

The effects of CAMs on binding angles could be more completely incorporated by making the preferred dimer-dimer binding angle and/or dihedral angle change upon CAM binding. However, the effects of CAM binding on subunit angles in the atomistic simulations of complete HBV capsids are limited by constraints arising from the neighboring subunits, and the resulting changes in the coarse-grained binding angles are small. Moreover, the remarkable flexibility of HBV dimers [37, 38] suggests that they may sample alternative conformations when not part of a capsid. Thus, accurately parameterizing the effects of CAMs on binding angles will require data from all-atom simulations of smaller intermediates in which fewer constraints from the capsid geometry are present, and potentially adding additional degrees of freedom to the coarse-grained model. However, these steps are beyond the scope of the current work.

### C. Outlook

The computational framework used in the present article can be readily generalized to other antiviral molecules or viruses, provided that atomistic simulation data and experimental data on binding affinities and rates is available for parameterization. For example, while this work was motivated by experiments looking at the effect of a particular CAM, HAP-TAMRA, on assembly, other CAMs have different effects on assembly [26, 62]. Moreover, researchers are investigating other potential antiviral molecules that perturb capsid assembly for a wide variety of viruses (e.g. [63-67]).

More broadly, our results elucidate the engineering principles that natural viruses have evolved to optimize their assembly, and how assembly pathways can be redirected to alternative structures. These insights could advance efforts in the emerging field of “synthetic structural biology” [68] to design de novo subunits that efficiently self-assemble into large, complex, and functional structures. For example, our results show that the binding of effector molecules to assembly subunits could be a powerful strategy for manipulating assembly morphologies. This strategy could be implemented synthetically by designing multicomponent assembly systems, e.g. using DNA origami [69-72] or protein design [73-79], in which small DNA strands [80] or peptides [81, 82] bind preferentially to the interfaces between certain components, perhaps stabilizing local geometries consistent with different assembled structures (e.g. nanotubes with different curvatures) [83]. Such synthetic systems could provide an alternative, controllable avenue for understanding the principles by which effector molecules stabilizing certain subunit conformations can control self-assembly.

## IV. METHODS

### A. Model

Our coarse-grained model for HBV assembly with CAMs extends the elastic shell growth model developed in Ref. [35, 43] for HBV assembly in the absence of CAMs. As described in Section II, capsid protein dimers (subunits) are represented as edges in a flexible triangular mesh (Fig. 1Ai,ii,iii). We characterize the mesh geometry by its edge lengths l, binding angles θ between two bound edges, and dihedral angles ϕ between pairs of triangles that share an edge. Each edge can switch between two conformations — AB and CD/CC — where A, B, C, and D refer to the four quasi-equivalent monomer conformations appearing in T=4 HBV capsids. The CD/CC designation refers to the fact that T=4 capsids have AB and CD asymmetric dimers whereas T=3 icosahedra have AB asymmetric dimers and CC symmetric dimers. When referring to a dimer-dimer interface, we only list the two monomers that are in contact; thus, e.g., the interface between a DC-DC dimer of dimers is referred to as the C-D interface. The lengths, binding angles, and dihedral angles fluctuate about their rest values, which depend on the conformations of the dimers involved (see Table I); deviations from the rest values incur an elastic energy cost.

In atomistic protein structures, CAMs such as HAP-TAMRA are bound to dimer-dimer interfaces (Fig. 1B, left). In our model, we represent CAMs as single beads that bind to the corners of the triangles (which represent dimer-dimer interfaces; Fig. 1B, right). Importantly, when two dimers bind, one acts as a “pocket” and the other acts as a “cap” [40]; thus, e.g., C-D and D-C interfaces are distinct. We represent this asymmetry in our model by using a directed half-edge data structure (see SI Section S2 A for details). Cryo-EM data indicate [36] that HAP-TAMRA – the CAM used in the experiments that inspired this work – primarily binds to quasi-sixfold vertices (i.e. B-C and C-D interfaces). However, the experiments of Kondylis et al. [12] suggest CAM binding favors flatter structures, and previous simulations by Mohajerani et al. suggest that extended, flat structures are composed primarily of dimers in the CD conformation. Motivated by these observations and the hypothesis that CAM binding to B-C interfaces is not essential for malformed assemblies (supported by atomistic simulations showing that B-C interfaces are “CAM-ready” and hence little prone to inducing structural distortions in capsids [39]), we reduce the number of parameters by considering a model in which CAMs bind only to only to C-D interfaces (found in T=4 capsids) and D-C interfaces (present in sheet-like structures, but not T=4 or T=3 capsids). The model could be easily extended to allow for binding to other interfaces as well.

We use a kinetic Monte Carlo (KMC) algorithm to simulate the dynamics of the mesh. We work in the grand canonical ensemble, where chemical potentials $\mu_{\text{dimer}}$ and $\mu_{\text{CAM}}$ set the concentrations of dimers and CAMs, respectively, in a reservoir. Since the concentrations are constant in our simulations, we simulate dynamics over time periods in the reaction which are short in comparison to the depletion of subunits or CAMs. The concentrations of CD and AB dimers in the reservoir follow from mass conservation and by the noting that the conformational free energy difference determines the ratio of their concentrations in the reservoir: $\left[\text{AB}\right]∕[\text{CD}]=\text{exp}(-\Delta g_{\text{CD}\to \text{AB}}^{\text{conf}}∕k_{B}T)$. Starting from an initial, three-dimer nucleus (Fig. 1Aiii, left), assembly proceeds by the addition of edges or pairs of edges, which represent the binding of dimers or dimers-of-dimers to the growing structure. Simultaneously, CAMs can bind/unbind to/from the corners of the triangles. Importantly, dimer-dimer and CAM-dimer-interface binding are reversible, and the relative rates of binding and unbinding are set by the dimer-dimer and CAM-dimer-interface binding free energies. We describe the model parameters and the KMC algorithm in more detail in the following subsections and in the SI.

The T=4 capsid corresponds to a free energy minimum in our model, which is the global minimum under most conditions that we simulate, except with CAMs at low salt concentration where sheets are favored (I=80mM; see SI Section S3). Fig. 1C illustrates the atomistic and coarse-grained structures of the complete capsid. The atomistic structure has HAP-TAMRA bound to B-C and C-D interfaces; the coarse-grained structure has CAMs bound only to the C-D interfaces. The rest values of dihedral angles (i.e. the preferred angle between the two triangles in each diamond) are such that the T=4 capsid minimizes the elastic energy. However, as shown by Mohajerani et al. [35], the diamonds are sufficiently flexible that T=3 capsids can also assemble (Fig. 1D). Additionally, CAMs can stabilize diamonds that consist only of CD dimers (Fig. 1E, right). These diamonds contain both C-D interfaces (present in T=4 capsids) and D-C interfaces (absent from T=4 capsids), and have a rest dihedral angle of zero. This stabilizes flat, non-capsid (“malformed”) structures, which we refer to as “sheets” (Fig. 1E, left).

### B. Units

We set σ=8nm as our unit of length, $t_{0}\;=\;10^{-5}\text{sec}∕\text{sweep}$ as our unit of time (where a sweep consists of a certain number of attempted Monte Carlo moves, as defined in Subsection C below), and $k_{B}T$ as our unit of energy, where $k_{B}\approx 1.381\times 10^{-23}\text{kg}{\left(m∕s\right)}^{2}∕K$ is Boltzmann’s constant and T=300K is the temperature in Kelvin. We express all other quantities in terms of σ, $t_{0}$ and $k_{B}T$.

### C. Coarse-grained energy function

The KMC dynamics obey detailed balance with respect to a coarse-grained energy function:

(1) $${ℋ}_{\text{assembly}}={ℋ}_{\text{elastic}}+{ℋ}_{\text{steric}}+{ℋ}_{\text{conf}}+{ℋ}_{\text{bind}}+{ℋ}_{\text{CAM}}$$

where the terms on the right-hand side account for, respectively, elastic, steric (excluded volume), conformational, dimer-dimer interaction, and dimer-CAM interaction free energies. The elastic energy function is:

(2) $${ℋ}_{\text{elastic}}={ℋ}_{\text{stretch}}+{ℋ}_{\text{bend}}+{ℋ}_{\text{dihedral}},$$

where the stretching, bending, and dihedral elastic energies are given respectively by:

(3) $${ℋ}_{\text{stretch}}=\sum_{\text{edges}}\frac{\kappa_{l}}{2}{\left(l-l_{c}^{0}\right)}^{2}$$

(4) $${ℋ}_{\text{bend}}=\sum_{\text{angles}}\frac{\kappa_{\theta }}{2}{\left(\theta -\theta_{c\text{-}c’}^{0}\right)}^{2}$$

(5) $${ℋ}_{\text{dihedral}}=\sum_{\text{dihedrals}}\kappa_{\phi }(1-\cos\;(\phi -\phi_{c,s}^{0}))$$

Here, l is the (instantaneous) length of an edge, θ is the angle between two edges within a triangle, and ϕ is the dihedral angle between two triangles; $l_{c}^{0}$ and $\theta_{c\text{-}c’}^{0}$ are the rest length and rest angle, whose values depend upon the conformations c of the edges involved (see Table I in the Appendix); and $\kappa_{l}=4200k_{B}T∕\sigma^{2}$, $\kappa_{\theta }=800k_{B}T$, and $\kappa_{\phi }=40k_{B}T$ are elastic moduli, whose values are inferred from atomistic molecular dynamics simulations of intact capsids [35]. (Note that to simplify notation, we write e.g. $\theta_{c\text{-}c’}^{0}=\theta_{A\text{-}A}^{0}$ when c=BA, $c^{\prime }=\mathrm{AB}$.) The quantity $\phi_{c,s}^{0}$ is the rest dihedral angle, which depends on the conformation c of the interior edge of the diamond that forms the dihedral and the conformations of the other edges in the diamond. Although the complete energy function depends on the conformations of all five dimers involved in a dihedral, we simplify the presentation in the text by writing s=capsid, malformed. Here “capsid” denotes diamonds that are present in T=3 and T=4 capsids and “malformed” refers to all other diamonds (which are found in mis-assembled structures). The most common malformed diamonds formed in our simulations are those that consist only of CD dimers (i.e. Fig. 1E).

The steric energy function, which enforces dimer excluded volume, is:

(6) ℋsteric=∑excluder pairs(i,j){∞,∣ri−rj∣<(σi+σj)∕20,otherwise,}

where $\sigma_{i}$ is diameter of a hard sphere “excluder” pseudoatom. There are four excluder pseudoatoms per dimer (edge), with three evenly spaced along the edge and one above the edge, the latter representing the dimer “spike” (see Fig. 1Aii). We set $\sigma_{i}=0.2\sigma$ for all excluders i.

The conformational energy function ${ℋ}_{\mathrm{conf}}$ accounts for the intrinsic free energy difference between different quasi-equivalent conformations $\Delta g_{\mathrm{CD}\to \mathrm{AB}}^{\mathrm{conf}}$, which is related to their relative concentrations at equilibrium by $K_{\mathrm{AB}}\equiv \frac{\left[AB\right]}{\left[CD\right]}=e^{-\Delta g_{\mathrm{CD}\to \mathrm{AB}}^{\mathrm{conf}}∕k_{B}T}$. Given their high degree of structural similarity, we set the CD and CC conformations to have the same intrinsic free energy ($K_{\mathrm{CC}}\equiv \frac{\left[CC\right]}{\left[CD\right]}=1$), and we set the CD/CC dimer as the reference conformation with zero conformational free energy. Hence, only the conformational free energy of AB dimers relative to CD/CC dimers, $\Delta g_{\mathrm{CD}\to \mathrm{AB}}^{\mathrm{conf}}$, appears in ${ℋ}_{\mathrm{conf}}$:

(7) $${ℋ}_{\text{conf}}=n_{\text{AB}}\Delta g_{\text{CD}\to \text{AB}}^{\text{conf}},$$

where $n_{\mathrm{AB}}$ is the number of AB dimers in the assembly. Although the conformational free energy difference $\Delta g_{\mathrm{CD}\to \mathrm{AB}}^{\mathrm{conf}}$ has not been directly estimated from experiments, the results of Mohajerani et al. [35] suggest that $\Delta g_{\mathrm{CD}\to \mathrm{AB}}^{\mathrm{conf}}$ decreases with increasing ionic strength. Consistent with their observations, we vary $\Delta g_{\mathrm{CD}\to \mathrm{AB}}^{\mathrm{conf}}$ from $5.5k_{B}T$ to $3.5k_{B}T$ with increasing ionic strength (see Table I).

The dimer-dimer binding energy function is:

(8) $${ℋ}_{\text{bind}}=\sum_{\text{bound edges}\;(i,j)}g_{c(i)\text{-}c(j)},$$

where the binding affinity (free energy) $g_{c\text{-}c^{\prime }}$ depends on the conformations c, $c^{\prime }$ of the two bound dimers, with values for specific pairs of conformations given in Table I. (To simplify notation, we write e.g. $g_{c\text{-}c^{\prime }}=g_{A\text{-}A}$ when c=BA, $c^{\prime }=\mathrm{AB}$.) These values were estimated from the buried surface area computed with PDBePISA [84, 85], as reported in Ref. [35]. We report values of $g_{c\text{-}c^{\prime }}$ for each possible interface, relative to a reference value $g_{0}^{\text{bind}}=g_{C\text{-}D}$ which varies weakly with salt concentration [35], in Table I. Note that the value of $g_{D\text{-}C}$ ($=0.5g_{0}^{\text{bind}}$) is larger than that used in Mohajerani et al. [35] ($0.2g_{0}^{\text{bind}}$). While they found that results were insensitive to the values of $g_{D\text{-}C}$ in this range, the slightly larger value better reproduces experimental size distributions in the presence of CAMs (Fig. 2) since increasing $g_{D\text{-}C}$ helps stabilize sheets (indeed, Ref. [35] found that setting $g_{C\text{-}D}\approx g_{0}^{\text{bind}}$ resulted in sheets forming without CAMs). Note also that, unlike in Ref. [35], the values reported in Table I do not account for the entropy of binding; this is instead accounted for via a binding volume, which is built into the KMC algorithm (see SI section S2 B). Together, $g_{0}^{\text{bind}}$ and $\Delta g_{\mathrm{CD}\to \mathrm{AB}}^{\mathrm{conf}}$ determine the mean dimer-dimer binding affinity within a T=4 capsid [35]:

(9) $$g_{T=4}^{\text{bind}}=\left(4.3g_{0}^{\text{bind}}+\Delta g_{\text{CD}\to \text{AB}}^{\text{conf}}\right)∕4,$$

where the factor of 4.3 comes from summing over the energies of the four different contacts present in equal numbers in a T=4 capsid (see Table I). Consistent with experiments, the magnitude of $g_{T=4}^{\text{bind}}$ increases with increasing salt concentration (see Table I). We reiterate that, in contrast to Ref. [35], we have not included the binding entropy in these values. We discuss simulation results (Section II) in terms of $g_{T=4}^{\text{bind}}$ and $\Delta g_{\mathrm{CD}\to \mathrm{AB}}^{\mathrm{conf}}$. Finally, the CAM binding energy function is:

(10) $${ℋ}_{\text{CAM}}=\sum_{\begin{array}{c} \text{bound edges}\;(i,j)\\ w∕\;\text{bound CAM} \end{array}}g_{c(i)\text{-}c(j)}^{\text{CAM}}$$

where $g_{c\text{-}c^{\prime }}^{\mathrm{CAM}}$ is the binding affinity of CAMs to dimer-dimer interfaces. In our model, CAMs bind only to C-D and D-C interfaces, and hence only $g_{C\text{-}D}^{\mathrm{CAM}}$ and $g_{D\text{-}C}^{\mathrm{CAM}}$ are defined (see Table I). Based on experimental binding affinities of HAP-ALEX (another fluorophore-labeled HAP) to capsids [61], we set $g_{C\text{-}D}^{\mathrm{CAM}}=-13k_{B}T$. No experimental data exists for binding to D-C interfaces because they do not occur in capsids. We choose $g_{D\text{-}C}^{\mathrm{CAM}}=g_{C\text{-}D}^{\mathrm{CAM}}+g_{C\text{-}D}-g_{D\text{-}C}$, which makes the free energy of a CAM-bound D-C interface equal to that of a CAM-bound C-D interface: $g_{C\text{-}D}+g_{C\text{-}D}^{\mathrm{CAM}}=g_{D\text{-}C}+g_{D\text{-}C}^{\mathrm{CAM}}$ (see SI section S2 A).

### D. Kinetic Monte Carlo (KMC) Move Set

We use a KMC algorithm consisting of nine different types of moves to simulate the assembly dynamics. These moves account for association and dissociation of both (1) single dimers and (2) dimers-of-dimers, (3,4,5) binding and unbinding of dimers within an assembly, which can occur in three topologically distinct ways, (6) dimer conformational switching, binding and unbinding of (7) CAMs and (8) CAM-bound dimers-of-dimers, and (9) thermal fluctuations of the mesh, accomplished via vertex displacement moves (see Figs. S8, S9, and SI Section S2 D for details). KMC moves are proposed with frequencies that reflect the rate constants of each process. A sweep consists of $n_{\mathrm{vert}}$ attempts to displace a randomly selected vertex, with $n_{\mathrm{vert}}$ the number of vertices in the assembly, and $n_{\mathrm{dimer}}$ attempts to change the conformation of a randomly selected edge, where $n_{\mathrm{dimer}}$ is the number of dimers/edges in the assembly. A sweep sets the timescale of our simulations, $t_{0}=10^{-5}\sec∕\text{sweep}$. We choose this value based on experimental measurements of HBV capsid assembly timescales and previous work [35, 86, 87]. Dimer association/dissociation moves are proposed at a rate $k_{0}^{\mathrm{dimer}}=2\times 10^{-2}∕t_{0}$, while CAM association/dissociation moves are proposed at a rate $k_{0}^{\mathrm{CAM}}=2\times 10^{-5}∕t_{0}$. The rate of CAM binding relative to dimer-dimer binding, $k_{0}^{\mathrm{CAM}}∕k_{0}^{\mathrm{dimer}}=10^{-3}$, is based on experimental kinetics measurements of HAP-ALEX binding to T=4 HBV capsids [61]. Detailed descriptions of each type of KMC move are given in SI Section S2 D.

### E. Simulation protocol

Each simulation begins with an initial triangular “nucleus” consisting of two AB dimers and one CD dimer as in Ref. [35]. We subsequently run KMC dynamics for a maximum of 2 × 10^8^ sweeps, where we define one sweep as $n_{\mathrm{dimer}}$ attempted vertex displacement moves. Additionally, simulations end when (i) a complete T=4 or T=3 capsid forms, (ii) assemblies grow to more than 240 edges (twice the size of a T=4 capsid), or (iii) the growth rate of a sufficiently large assembly becomes sufficiently slow (see SI Section S2 C for details). We run 100 independent KMC simulations (using different, randomly-generated, random seeds) for each value of ionic strength, and both with and without CAMs.

### F. Markov state model

To construct a Markov state model (MSM), one chooses a discretization scheme that partitions microscopic configurations into M different ‘microstates,’ such that transitions between microstates are Markovian. Given a collection of many trajectories, one then counts the number of transitions into and out of each microstate after a certain lag time (τ) to obtain an M×M count matrix, C, with elements $C_{ij}$ representing the number of transitions from microstate i to microstate j separated by a time τ. Column-normalizing the count matrix gives the transition matrix T(τ), with elements given by $T_{ij}=C_{ij}∕\sum_{j}C_{ij}$. The matrix elements $T_{ij}$ then give the rates of transitioning from microstate i to microstate j. We partitioned configurations from our KMC trajectories into microstates characterized by the number of AB dimers ($n_{\mathrm{AB}}$), the number of T=4-like CD dimers ($n_{\mathrm{CD}}^{T=4}$), and the number of sheet-like CD dimers ($n_{\mathrm{CD}}^{\text{sheet}}$). A sheet-like CD dimer forms an edge shared by two triangles (i.e. it is the “central” edge of a diamond), both of which consist solely of CD dimers (see e.g. Fig. 1E). A T=4-like CD dimer forms an edge that is shared by a triangle consisting of only CD dimers and a triangle with two AB dimers and one CD dimer; see Fig. 1Aiii, right.

Given the transition matrix, we can compute the time-dependent probability vector p(t) as:

(11) $$p(n\tau )={\left(T(\tau )\right)}^{n}p(0),$$

where each element $p_{i}(t)$ gives the probability of being in microstate i at time t, and time is advanced in multiples of the lag time, t=nτ. We performed a standard test (see SI Section S4 and Fig. S10) to confirm that the MSM dynamics are indeed Markovian for large enough τ; we chose τ=0.02s. As a more stringent test, we also confirmed that the MSM reproduces KMC dynamics: specifically, the MSM yields as a function of time for T=4 capsids and sheets agree well with those obtained from brute-force KMC simulations (Fig. S11).

### G. Committor probabilities

The committor $q_{i}^{B}$ is the conditional probability of reaching a state (or set of states) B before reaching another state (or set of states) A, given that the system starts in state i [52, 53]. For an MSM with transition matrix T, the committor is given by the following equation [52, 53]:

(12) $$q_{i}^{B}-\sum_{j\in I}T_{ij}q_{j}^{B}=\sum_{j\in B}T_{ij},$$

where I denotes the set of intermediate states, i.e. those states which are neither in B nor in A. We compute committors for T=4 assembly ($q_{i}^{T=4}$) and sheet assembly ($q_{i}^{\text{sheet}}$). For T=4, state B is the microstate with $n_{\mathrm{AB}}=60$, $n_{\mathrm{CD}}^{T=4}=60$, and $n_{\mathrm{CD}}^{\text{sheet}}=0$. For sheets, state B consists of all microstates with $n_{\mathrm{CD}}^{T=4}\geq 10$. In both cases, state A consists of the initial three-dimer nucleus and all absorbing states which are not in state B. Eq. 12 is a linear system, which we solve via least squares.

### H. Visualization

Both atomistic structures and coarse-grained simulation snapshots were created using Visual Molecular Dynamics [88].

## Supplementary Material

Supplement 1

Supplement 2

## Data Availability

Code for running simulations, as well as code and data for reproducing figures, is available on Zenodo at: [link to be inserted upon publication]

## Appendix A: Simulation parameters

Table I lists the model parameters, their values, and brief descriptions of them.

**TABLE I.** Simulation parameter values and descriptions.

Salt-dependent interaction and thermodynamic parameters		
Parameter	Value(s)	Description
$\Delta g_{\mathrm{CD}\to \mathrm{AB}}^{\mathrm{conf}}(k_{B}T)$	{3.5,I=1000mM4.5,I=300mM5.5,I=80mM}	conformational free energy difference between CD and AB dimers
$g_{0}^{\text{bind}}(k_{B}T)$	{−9.76,I=1000mM−9.80,I=300mM−9.85,I=80mM}	reference dimer-dimer binding free energy
$g_{T=4}^{\text{bind}}(k_{B}T)$	{−9.62,I=1000mM−9.42,I=300mM−9.22,I=80mM}	mean dimer-dimer binding free energy in T=4 capsid
Relative interdimer binding affinities (estimated from PDBePISA, Refs. [35, 84, 85]), with $g_{D\text{-}C}$ adjusted for assembly with CAMs)		
Parameter	Value(s)	Description
$g_{A\text{-}A}∕g_{0}^{\text{bind}}$	1.3	A-A binding free energy
$g_{B\text{-}C}∕g_{0}^{\text{bind}}$	1.1	B-C binding free energy
$g_{C\text{-}D}∕g_{0}^{\text{bind}}$	1	C-D binding free energy
$g_{D\text{-}B}∕g_{0}^{\text{bind}}$	0.9	D-B binding free energy
$g_{D\text{-}C}∕g_{0}^{\text{bind}}$	0.5	D-C binding free energy
CAM interaction parameters		
Parameter	Value(s)	Description
$g_{C\text{-}D}^{\mathrm{CAM}}(k_{B}T)$	−13	binding free energy of CAM to DC-DC interface
$g_{D\text{-}C}^{\mathrm{CAM}}(k_{B}T)$	−13 + ($g_{C\text{-}D}-g_{D\text{-}C}$)	binding free energy of CAM to CD-CD interface
Chemical potentials and rate constants		
Parameter	Value(s)	Description
$\mu_{\text{dimer}}(k_{B}T)$	−11.5	dimer chemical potential (corresponds to ≈ 10μM)
$\mu_{\mathrm{CAM}}(k_{B}T)$	−10.1	CAM chemical potential (corresponds to ≈ 40μM)
$k_{0}^{\mathrm{dimer}}(t_{0}^{-1})$	2 × 10^−2^	relative dimer binding rate (ratio of attempted dimer binding moves to attempted vertex moves)
$k_{0}^{\mathrm{CAM}}(t_{0}^{-1})$	2 × 10^−5^	relative CAM binding rate (ratio of attempted CAM binding moves to attempted vertex moves)
Elastic and geometric parameters (derived from all-atom simulations and capsid geometry, following Ref. [35])		
Parameter	Value(s)	Description
$\kappa_{l}(k_{B}T∕\sigma^{2})$	4200	bond stretching modulus
$\kappa_{\theta }(k_{B}T)$	800	binding angle bending modulus
$\kappa_{\phi }(k_{B}T)$	40	dihedral angle bending modulus
$\sigma_{i}(\sigma )$	0.2	excluder diameter
$l_{\mathrm{AB}}^{0}(\sigma )$	0.95	AB dimer rest length
$l_{\mathrm{CD}}^{0}(\sigma )$	1.05	CD dimer rest length
$\theta_{A\text{-}A}^{0}(\mathrm{rad})$	1.17	rest angle between BA and AB dimers (A-A interface)
$\theta_{B\text{-}C}^{0}(\mathrm{rad})$	0.98	rest angle between AB and CD(CC) dimers (B-C interface)
$\theta_{D\text{-}B}^{0}(\mathrm{rad})$	0.98	rest angle between CD(CC) and BA dimers (D-B interface)
$\theta_{C\text{-}D}^{0}(\mathrm{rad})$	1.05	rest angle between DC and DC dimers (C-D interface)
$\theta_{D\text{-}C}^{0}(\mathrm{rad})$	1.05	rest angle between CD and CD dimers (D-C interface)
$\phi_{\mathrm{AB},\text{capsid}}^{0}(\mathrm{rad})$	0.48	rest dihedral with AB interior edge in capsid
$\phi_{\mathrm{CD},\text{capsid}}^{0}(\mathrm{rad})$	0.24	rest dihedral with CD interior edge in capsid
$\phi_{\mathrm{CD},\text{malformed}}^{0}(\mathrm{rad})$	0	rest dihedral with CD interior edge in malformed structure (e.g. sheet)
