# Supplementary material for: Modeling Reveals How Direct-Acting Antivirals Redirect HBV Capsid Assembly Pathways to Noninfectious Products
Source: bioRxiv. 2026 May 26:2026.05.25.727729. Preprint. [Version 1] doi: 10.64898/2026.05.25.727729 (PMC13232356; doi:10.64898/2026.05.25.727729)
Supplement: Supplement 2 [file NIHPP2026.05.25.727729v1-supplement-2.pdf]

bioRxiv preprint doi: https://doi.org/10.64898/2026.05.25.727729; this version posted May 26, 2026. The copyright holder for this preprint (which was not certified by peer review) is the author/funder, who has granted bioRxiv a license to display the preprint in perpetuity. It is made available under a CC-BY 4.0 International license.

Supplementary material: Modeling Reveals How Direct-Acting Antivirals Redirect HBV Capsid Assembly Pathways to Noninfectious Products

Layne B. Frechette,[1,*] Smriti Pradhan,[1,*] Farzaneh Mohajerani,[1] Carolina Pérez-Segura,[2] Jodi A. Hadden-Perilla,[2] Adam Zlotnick,[3] and Michael F. Hagan[1,†]

[1] *Martin Fisher School of Physics, Brandeis University, Waltham, Massachusetts 02453, USA*
[2] *Department of Chemistry & Biochemistry, University of Delaware, Newark, Delaware 19716, USA*
[3] *Molecular and Cellular Biochemistry Department, Indiana University, Bloomington, Indiana 47405, USA*

(Dated: May 15, 2026)

CONTENTS

S1. Additional Figures 1

S2. Model 6
  A. Implementation of the coarse-grained (CG) model 6
  B. Monte Carlo (MC) Simulations 7
  C. Shell assembly simulations 8
  D. MC moves 8

S3. Free energies of capsids and sheets 14

S4. Markov state model validation 15

S5. Movie descriptions 17

References 17

S1. ADDITIONAL FIGURES

* These two authors contributed equally.

† hagan@brandeis.edu

bioRxiv preprint doi: https://doi.org/10.64898/2026.05.25.727729; this version posted May 26, 2026. The copyright holder for this preprint (which was not certified by peer review) is the author/funder, who has granted bioRxiv a license to display the preprint in perpetuity. It is made available under a CC-BY 4.0 International license.

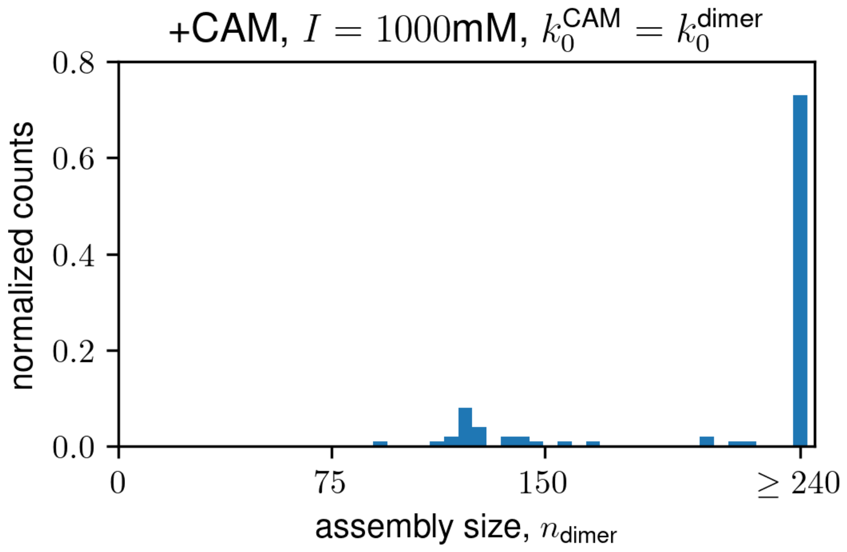

FIG. S1. **Fast CAM binding yields malformed structures at high salt concentrations.** Assembly size distribution from simulations with CAMs at $I = 1000$mM, in which the CAM binding rate constant has been increased from $k_0^{\text{CAM}}/k_0^{\text{dimer}} = 10^{-3}$ to $k_0^{\text{CAM}}/k_0^{\text{dimer}} = 1$. Roughly 70% of the trajectories end in large malformed structures with $n_{\text{dimer}} \geq 240$.

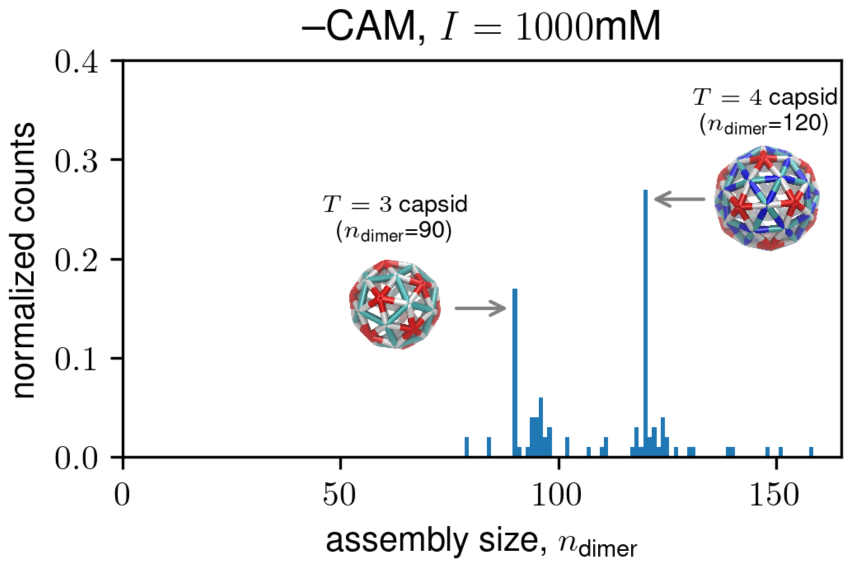

FIG. S2. **Without CAMs, $T = 3$ capsids occur at high salt concentrations.** Assembly size distribution without CAMs at $I = 1000$mM. There are two large peaks at $n_{\text{dimer}} = 90$, corresponding to $T = 3$ capsids, and at $n_{\text{dimer}} = 120$, corresponding to $T = 4$ capsids. Snapshots show the coarse-grained structures of $T = 3$ and $T = 4$ capsids.

bioRxiv preprint doi: https://doi.org/10.64898/2026.05.25.727729; this version posted May 26, 2026. The copyright holder for this preprint (which was not certified by peer review) is the author/funder, who has granted bioRxiv a license to display the preprint in perpetuity. It is made available under a CC-BY 4.0 International license.

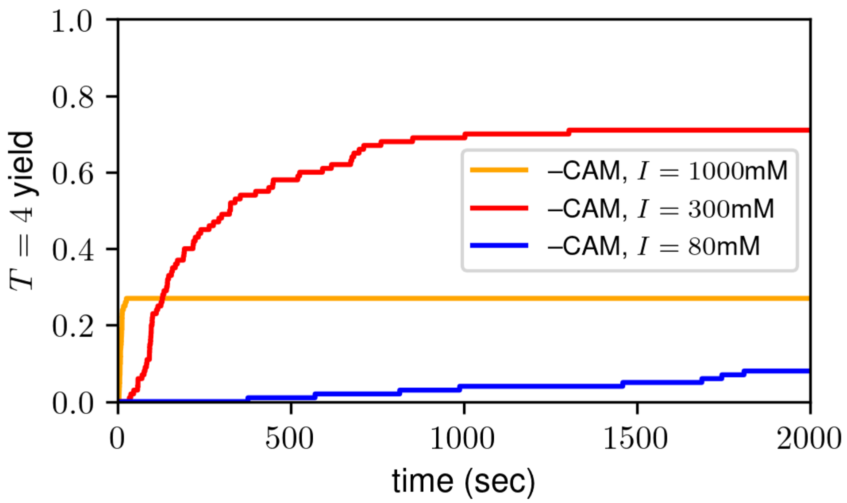

FIG. S3. **Without CAMs, $T = 4$ capsid formation rate increases with salt concentration.** $T = 4$ capsid yield (the fraction of trajectories that end in a $T = 4$ capsid) as a function of time for three different salt concentrations. As the ionic strength increases, the rate at which $T = 4$ capsids form also increases.

bioRxiv preprint doi: https://doi.org/10.64898/2026.05.25.727729; this version posted May 26, 2026. The copyright holder for this preprint (which was not certified by peer review) is the author/funder, who has granted bioRxiv a license to display the preprint in perpetuity. It is made available under a CC-BY 4.0 International license.

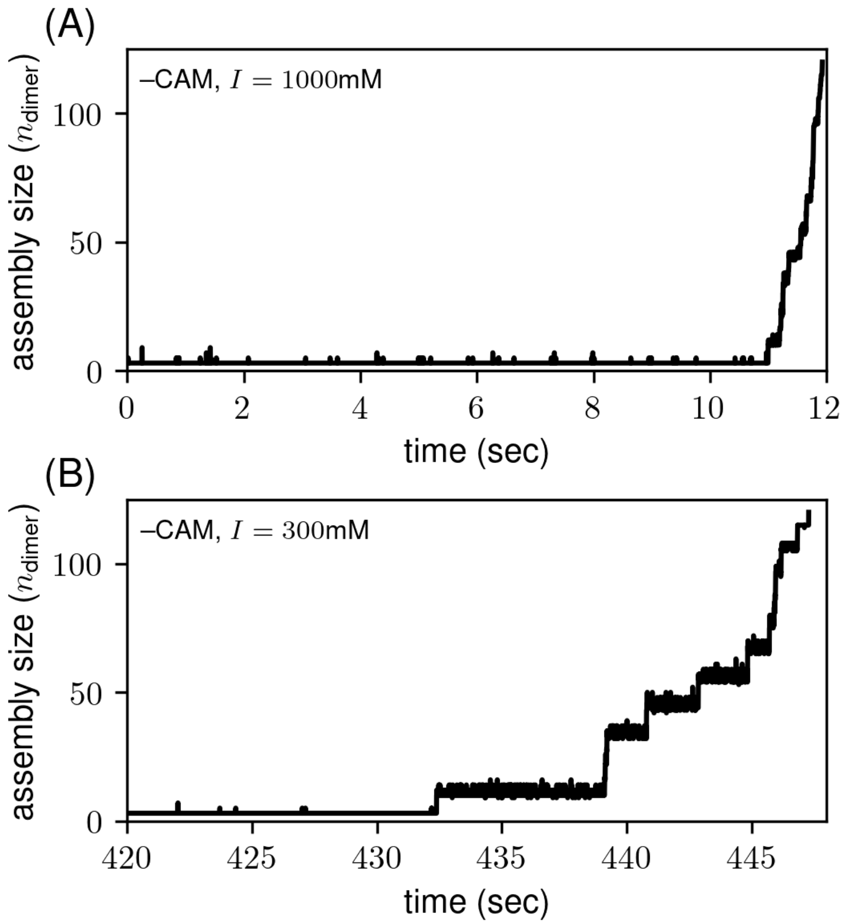

FIG. S4. **Trajectories leading to $T = 4$ capsids without CAMs are similar to those with CAMs.** Assembly size (in n_{dimer}) versus time for trajectories without CAMs at (A) $I = 1000$mM and (B) $I = 300$mM. Panel A shows the entire trajectory, while the plot in panel B starts at the beginning of the growth phase. These trajectories exhibit similar dynamics and intermediate sizes to the $T = 4$ trajectories with CAMs (Fig. 3 in the main text).

bioRxiv preprint doi: https://doi.org/10.64898/2026.05.25.727729; this version posted May 26, 2026. The copyright holder for this preprint (which was not certified by peer review) is the author/funder, who has granted bioRxiv a license to display the preprint in perpetuity. It is made available under a CC-BY 4.0 International license.

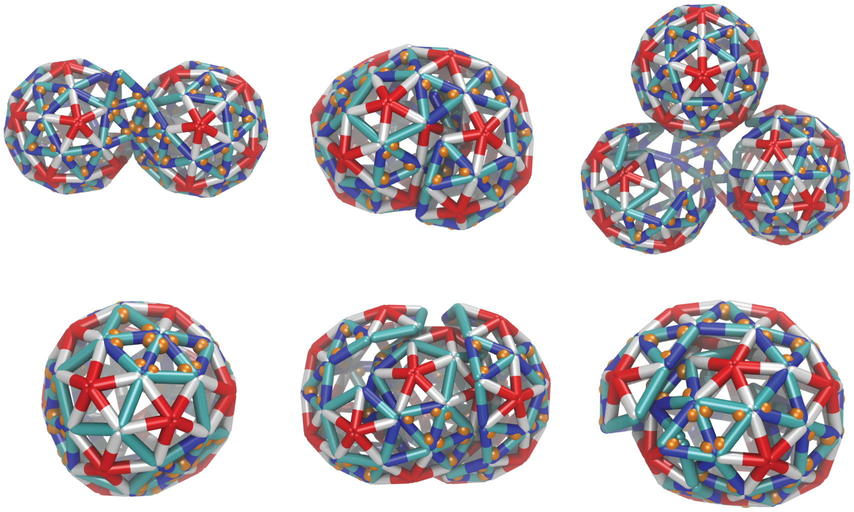

FIG. S5. **CAMs can induce malformed structures at high salt concentrations.** Representative snapshots of malformed structures with CAMs at $I = 1000$mM, including 'dumbbell' structures with two connected partial $T = 4$-like shells, 'triple dumbbell' structures with three connected partial $T = 4$-like shells, closed shells with non-$T = 4$ symmetry, and shells with overhangs.

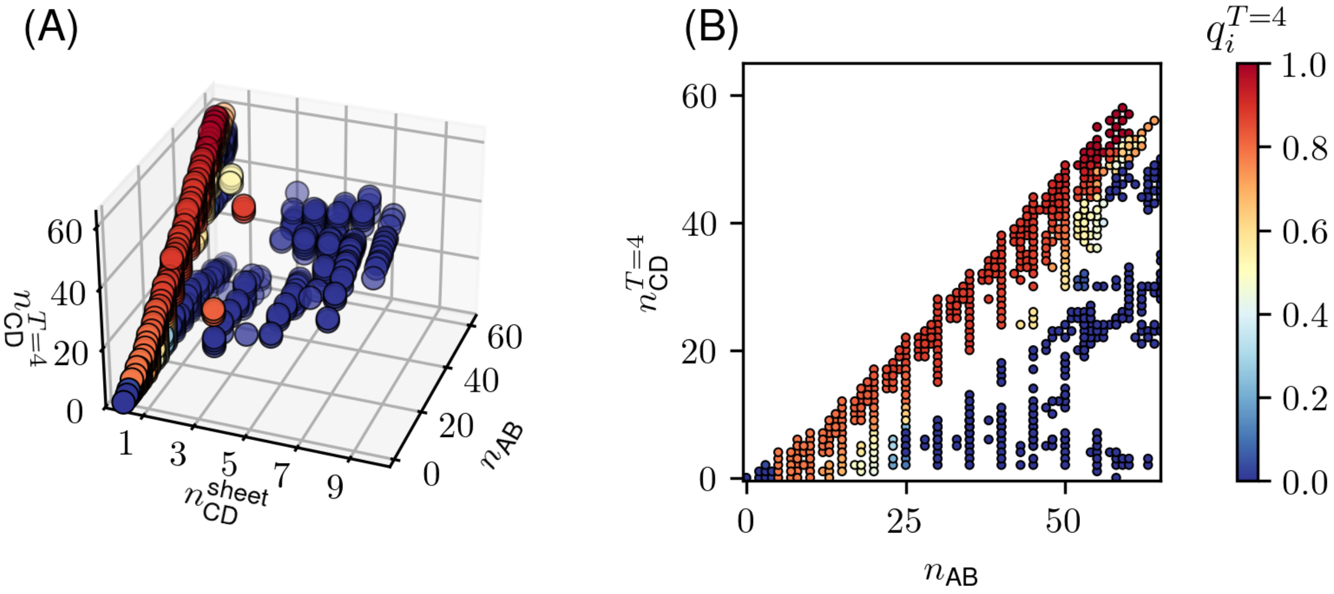

FIG. S6. **Committors reveal hub states for assembly without CAMs.** Committors $q_i^{T=4}$ for $T = 4$ capsid assembly (A) as a function of n_{AB}, $n_{\mathrm{CD}}^{T=4}$, and $n_{\mathrm{CD}}^{\mathrm{sheet}}$ and (B) as a function of n_{AB} and $n_{\mathrm{CD}}^{T=4}$ at $n_{\mathrm{CD}}^{\mathrm{sheet}} = 0$. Hub states (with $q_i^{T=4} \approx 0.5$) exhibit mixed $T = 4/T = 3$ morphologies (see main text Fig. 7D and Ref. [1]).

bioRxiv preprint doi: https://doi.org/10.64898/2026.05.25.727729; this version posted May 26, 2026. The copyright holder for this preprint (which was not certified by peer review) is the author/funder, who has granted bioRxiv a license to display the preprint in perpetuity. It is made available under a CC-BY 4.0 International license.

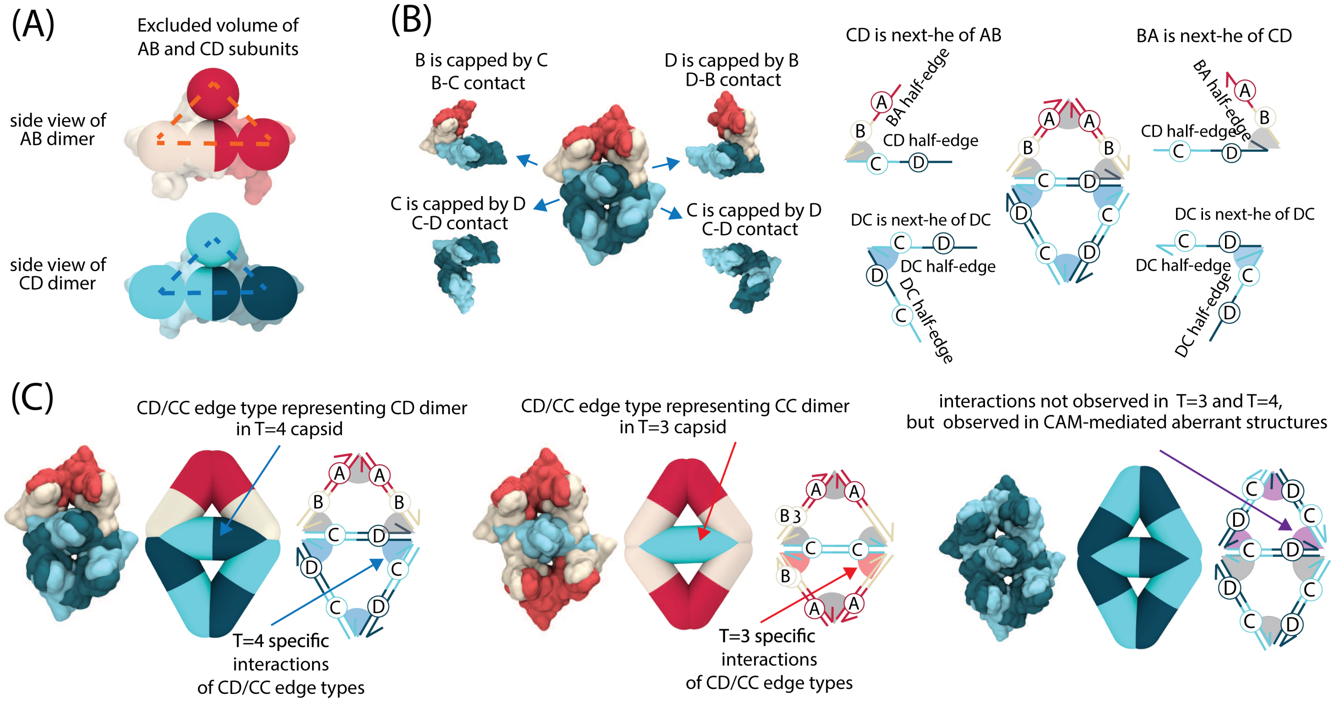

FIG. S7. **Mapping the half-edge data structure to HBV dimers.** **(A)** Side view of overlay of HBV AB and CD dimers and model subunits with excluder pseudoatoms. Subunits are also prevented from overlapping with each other by forbidding the plane shown by dashed lines to intersect the corresponding planes on other subunits. **(B)** The four different contacts of the middle CD subunit in a T=4 intermediate structure (left) and the relevant implementation of each contact by the half-edge data structure (right). In an HBV capsid, each dimer is capped in two of its four contacts, in a specific order as shown in the figure, which is represented by the contacts of two oppositely directed half-edges within an edge. **(C)** The two edge types, AB and CD, are shown along with the different edge interactions that they can make, to represent different conformations and interactions of HBV capsid protein dimers that are observed in available structures: $T = 4$ (left), $T = 3$ (middle), and hexameric sheets (right). Adapted from Ref. [1], copyright 2022 ACS Publications.

S2. MODEL

Many of the details of our model were described in our previous publication [1]. However, for ease of reference, we have copied those details here, with minor edits. We also describe the implementation of CAMs in the model.

A. Implementation of the coarse-grained (CG) model

As described in our previous publication [1], we implement our model of dimer subunits using the half-edge data structure (HE) [2], which is a doubly connected edge list [3]. In our model, each edge corresponds to a protein dimer and consists of two half-edges. There are two edge types: The type AB edge consists of AB and BA half-edges pointing in opposite directions and represents the AB dimer conformation in T=3 and T=4 capsids; the type CD edge consists of CD and DC half-edges pointing in opposite directions and represents the CD dimer in T=4 capsids or the CC dimer in T=3 capsids (Fig. S7A,B). Each of these edge types has an equilibrium length l_c^0 with $c = \mathrm{AB}, \mathrm{CD}$ labeling the conformation, and each interior edge (located between two adjacent triangular faces in the structure) has an equilibrium dihedral angle $\phi_{c,s}^0$ ($s \in$ (capsid, malformed)). Due to the high structural similarity between CD and CC dimers, we implement them with the same values of l_c^0 and $\phi_{c,s}^0$ and thus associate them with a single edge type. Throughout the article a CD/CC dimer is called a CD dimer except when it has a (CC/DC)-BA or AB-(CC/DC) interaction (Fig. S7C), since these two interactions are observed in T=3 capsids but not in T=4 capsids.

The advantage of the half-edge data structure over other, related triangular sheet models [4, 5] is that the two half-edges allow different parameters for protein-protein interactions between different monomer conformations. In particular, with 4 half-edges $c \in \{\mathrm{AB}, \mathrm{BA}, \mathrm{CD}, \mathrm{DC}\}$, there are up to 16 different values for binding affinities $g_{c\text{-}c'}$ and

bioRxiv preprint doi: https://doi.org/10.64898/2026.05.25.727729; this version posted May 26, 2026. The copyright holder for this preprint (which was not certified by peer review) is the author/funder, who has granted bioRxiv a license to display the preprint in perpetuity. It is made available under a CC-BY 4.0 International license.

c(h)	c′(next-h)	contact	$g_{c\text{-}c'}/g_0^{\text{bind}}$	$\theta_{c,c'}$	structure
BA	AB	A-A	1.3	1.17	T4,T3
AB	CD(CC)	B-C	0.9	0.98	T4,T3
CD(CC)	BA	D-B	1.1	0.98	T4,T3
AB	DC(CC)	B-D	0.9	0.98	T3
DC(CC)	BA	C-B	1.1	0.98	T3
DC	DC	C-D	1	1.05	T4
CD	CD	D-C	0.5	1.05	w/CAM

TABLE S1. Interaction angles and the relative binding affinities for the different conformations of interacting dimer pairs observed in assembled structures.

	$g_{\text{A-A}}/k_\text{B}T$	$g_{\text{D-B}}/k_\text{B}T$	$g_{\text{B-C}}/k_\text{B}T$
6UI7 T=4 capsid [6]	-8.36	-7.35	-6.11
	$g_{\text{A-A}}/k_\text{B}T$	$g_{\text{C-B}}^{bind}/k_\text{B}T$	$g_{\text{B-C}}/k_\text{B}T$
6UI6 T=3 capsid [6]	-8.42	-7.71	-6.52

TABLE S2. Comparison of dimer-dimer binding affinities in T=4 and T=3 capsids.

equilibrium binding angles $\theta^0_{\text{c-c}'}$ for dimer-dimer interactions. Note that the edges are asymmetric, and thus $g_{c\text{-}c'}$ and $\theta^0_{\text{c-c}'}$ depend on the order of the two half-edges involved; i.e., in general $g_{c\text{-}c'} \neq g_{c'\text{-}c}$.

Table S1 shows the binding affinity matrix for the possible half-edge-interactions in the model. Each table entry shows the binding affinity (in units of the thermal energy, $k_\text{B}T$) for a pair of half-edges, each belonging to a different edge. The 'structure' column lists the structures in which each type of contact is observed. For interactions that involve CC dimers in T=3 capsids, CC dimers have similar buried surface area as the CD dimer in T=4 capsids in the corresponding local geometry; i.e., CC-BA and AB-CC interfaces in T=3 capsids have very similar buried surface areas as CD-BA and AB-DC interfaces in T=4 capsids (see Table S2 and reference [6]).

The rest angles $\theta^0_{\text{c-c}'}$ for dimer-dimer interactions observed in T=4 and T=3 structures were obtained from all-atom (AA) simulations of T=4 capsid, as explained in our previous work [1]. All other rest angles are set to $\pi/3$ since they generally occur in flat or nearly flat hexagonal structures. Similarly, the rest dihedral angles ϕ_c associated with interior edges observed in T=4 and T=3 structures observed in were obtained from AA simulations. All other equilibrium dihedral angles are set to 0, consistent with flat or nearly flat hexagonal structures.

We represent CAMs as single beads which bind to the interface between two dimers (i.e. where two half-edges in different edges meet). As stated in the main text, we set $g^{\text{CAM}}_{\text{D-C}} = g^{\text{CAM}}_{\text{C-D}} + (g_{\text{C-D}} - g_{\text{D-C}})$, which makes the free energy of a CAM-bound D-C interface equal to that of a CAM-bound C-D interface: $g_{\text{C-D}} + g^{\text{CAM}}_{\text{C-D}} = g_{\text{D-C}} + g^{\text{CAM}}_{\text{D-C}}$ In preliminary simulations where we set $g^{\text{CAM}}_{\text{D-C}} = g^{\text{CAM}}_{\text{C-D}}$, sheets would grow in a slow, step-like fashion, in which CD interfaces formed quickly but DC interfaces formed slowly and transiently (because $g_{\text{C-D}} \neq g_{\text{D-C}}$). This led to very slow sheet growth on accessible simulation timescales. Setting $g^{\text{CAM}}_{\text{D-C}} = g^{\text{CAM}}_{\text{C-D}} + (g_{\text{C-D}} - g_{\text{D-C}})$ instead resulted in much faster sheet growth and assembly product distributions qualitatively consistent with experiments.

B. Monte Carlo (MC) Simulations

Our grand canonical Monte Carlo simulation implementation is adapted from Refs. [1, 5], in which the triangular sheet is represented by edges and vertices. In our model, each edge is associated with two vertices, which together have 2×3 degrees of freedom. Any two bound edges in the shell share a vertex, and thus together have 3×3 degrees of freedom. For a shell with n_{dimer} edges and n_{CAM} bound CAMs, the grand canonical probability density is

$$P(n_{\text{dimer}}, \{n_{c,c'}\}, n_{\text{CAM}}, \mathbf{X}) = \prod_{c,c'} K_{c,c'}^{n_{c,c'}} \; z^{n_e} \; (K_{\text{CAM},c,c'} z_{\text{CAM}})^{n_{\text{CAM}}} \; e^{-\beta(\mathcal{H}_{\text{elastic}}(\mathbf{X}) + \mathcal{H}_{\text{steric}}(\mathbf{X}) + \mathcal{H}_{\text{conf}})} \;/\; Z \qquad \text{(S1)}$$

where $c \in$ {AB,BA,CD,DC} are the different half-edge types (representing their conformations and directions) and $n_{c,c'}$ is the number of interactions within the shell configuration between pairs of dimers with c and c' conformations respectively. $K_{c,c'} = v_\text{b} C_{\text{ss}} e^{-\beta \epsilon_{c,c'}}$, with $\epsilon_{c,c'}$ the binding affinity of interacting half-edges with conformations c and c', v_b the binding volume, and C_{ss} the standard state concentration. $z = e^{-\beta\mu}$ is the activity with the chemical potential $\mu = \log(C_{\text{tot}}/C_{\text{ss}})$ where C_{tot} is the dimer concentration in solution. The standard state concentration is set to $C_{\text{ss}} = 1\text{M}$. $K_{\text{CAM},c,c'} = e^{-\beta g^{\text{CAM}}_{c\text{-}c'}}$, and $z_{\text{CAM}} = e^{-\beta \mu_{\text{CAM}}}$. The term $\mathcal{H}_{\text{conf}}$ accounts for the conformational free energy difference of the two types of dimers in the shell. $\mathbf{X}$ denotes the positions of all the vertices in the sheet, $\mathcal{H}_{\text{elastic}}(\mathbf{X})$

bioRxiv preprint doi: https://doi.org/10.64898/2026.05.25.727729; this version posted May 26, 2026. The copyright holder for this preprint (which was not certified by peer review) is the author/funder, who has granted bioRxiv a license to display the preprint in perpetuity. It is made available under a CC-BY 4.0 International license.

accounts for the elastic energy penalty for deviations from the ground state of the sheet, and $\mathcal{H}_{\text{steric}}(\mathbf{X})$ accounts for excluded volume among dimers. Finally, the partition function Z ensures that the grand canonical probability density is normalized.

The binding volume $v_{\text{b}} = (2\pi)^{3/2}\sigma_{\text{th}}^3$, where $\sigma_{\text{th}} = \sqrt{3l_{\text{AB}}^2 k_{\text{B}}T/(2\kappa_\phi)}/2$ is a thermal length scale. The quantity $\log(v_{\text{b}}C_{\text{ss}})$ represents an entropic free energy penalty for dimer-dimer binding, effectively modifying the free energy $g_{c\text{-}c'}$ relative to its "bare" value $\epsilon_{c,c'}$, i.e. $g_{c\text{-}c'} = \epsilon_{c,c'} - k_{\text{B}}T\log(v_{\text{b}}C_{\text{ss}})$.

In each MC step, a trial Monte-Carlo move ν is chosen randomly from the list of Monte-Carlo moves (described below) according to its relative trial rate k_0^ν. The trial moves are accepted/rejected based on the Metropolis-Hastings algorithm, ensuring detailed balance.

C. Shell assembly simulations

To simulate capsid assembly dynamics, we intersperse a variety of MC moves, described in the next section, that are designed to capture the physical dynamics of assembling subunits. Each simulation begins with an initial state comprising three edges in a triangular face. This state is known to be a highly populated intermediate, and is estimated to be the critical nucleus for HBV capsid assembly [7–9]. Each simulation contains a single assembly, which undergoes exchange of subunits and CAMs with a reservoir according to the grand canonical probability density, Eq. (S1). The simulations are performed for a maximum of 2×10^8 sweeps, where a sweep is defined as a set of trial moves consisting of addition/removal, binding/unbinding, shell relaxation, and conformational switch moves such that each edge on average will have undergone one conformational switch move and each vertex on average will have undergone one vertex displacement move. Simulations are stopped early if the assembly forms a closed structure, defined as a structure in which every edge has its maximum number of four interactions. Simulations are also stopped early if the assembly becomes stalled in a sufficiently long-lived intermediate, defined as a structure with $n_{\text{dimer}} \geq 75$ edges which has grown by no more than two dimers in the previous $n_{\text{stall}} = 100/\alpha$ sweeps, where α is the average growth rate (difference between the number of dimers every 10^4 MC sweeps, averaged over subsequent intervals of 10^4 MC sweeps) in a given simulation after the structure has reached $n_{\text{dimer}} = 35$ edges.

D. MC moves

The set of MC moves and their acceptance criteria are described here. Moves are accepted or rejected according to the Metropolis-Hastings acceptance criteria [10, 11]:

$$\frac{P(\Gamma')}{P(\Gamma)} = \frac{p_{\text{gen}}(\Gamma \to \Gamma')\; p_{\text{acc}}(\Gamma \to \Gamma')}{p_{\text{gen}}(\Gamma' \to \Gamma)\; p_{\text{acc}}(\Gamma' \to \Gamma)} \tag{S2}$$

where Γ and Γ' denote the initial and trial states, the probability of a state with n_{dimer} edges and n_{CAM} CAMs is $P(\Gamma) = \frac{1}{Z}e^{-\beta(\mathcal{H}_{\text{capsid}} - n_{\text{dimer}}\mu - n_{\text{CAM}}\mu_{\text{CAM}})}$ with Z the grand canonical partition function, and p_{gen} and p_{acc} are respectively the probabilities for generating and accepting trial moves.

Notation. Figure S8A shows a schematic of an example structure, with explanations of some notation that will be used in the following descriptions of the MC moves. Edges which have their full complement of four interactions are denoted as *non-boundary edges*. The two half-edges within a non-boundary edge are denoted as *non-boundary half-edges* (black arrows in Figure S8A). Each non-boundary half-edge interacts with a half-edge on each of the two neighboring edges, which are denoted as *next-h* (from its head) and *prev-h* (from its tail). An example of a half-edge **h** with its next-h and prev-h is shown in Figure S8A.

A *boundary half-edge* has at least one unbound end; i.e., it lacks an interaction with at least one of its adjoining half-edges (next-h or prev-h). By keeping track of the set of boundary half-edges, the simulation algorithm is able to efficiently choose possible trial moves which involve binding new edges. In the MC implementation used for this work, we allow only one half-edge in each edge to be a boundary half-edge, which prevents formation of dangling edges (that have only one interaction) and star-like configurations. Any trial move that results in formation of an edge that comprises two boundary half-edges is rejected.

In the description of the MC moves that follows we describe moves in terms of changes in half-edges. However, note that in general each move affects both half-edges within an edge, and these effects occur simultaneously.

Vertex move: A vertex is randomly chosen, and a trial is made to displace it by the vector $\vec{\delta x}$, whose components are chosen from a Gaussian distribution $\mathcal{N}(0, \sigma_{\text{th}}^2)$. $\sigma_{\text{th}} = \sqrt{3l_{\text{AB}}^2 k_{\text{B}}T/(2\kappa_\phi)}/2$ is the length scale for the thermal fluctuations of the system. In this move, the numbers of dimers, CAMs, dimer-dimer and dimer-dimer-CAM interactions,

bioRxiv preprint doi: https://doi.org/10.64898/2026.05.25.727729; this version posted May 26, 2026. The copyright holder for this preprint (which was not certified by peer review) is the author/funder, who has granted bioRxiv a license to display the preprint in perpetuity. It is made available under a CC-BY 4.0 International license.

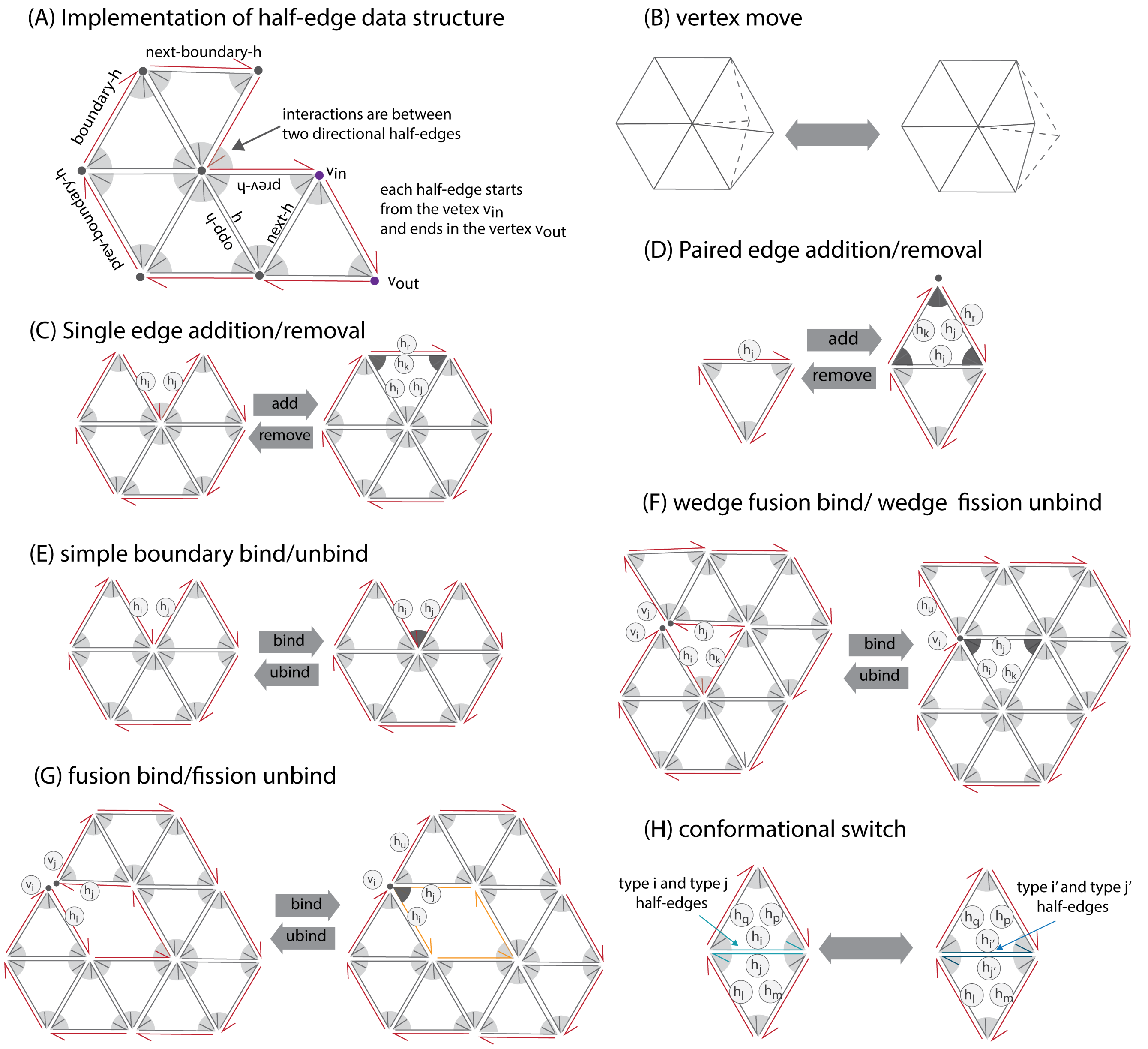

FIG. S8. **Schematic of the half-edge implementation and the MC moves. (A)** Half-edge implementation. Each edge in the model is composed of two half-edges. Half-edges with no open ends are designated as *non-boundary half-edges*, and drawn as grey arrows. For a compact notation, for a given half-edge, the half-edges that it interacts with on its tail and on its head are designated as *previous-h* and *next-h* respectively. Half-edges that are open (have no interaction partner) on their head and/or tail are designated as *boundary half-edges*, and drawn as red arrows. Each boundary half-edge has a *next-boundary-h* and a *prev-boundary-h*, but there is not necessarily an interaction between a half-edge and its next-boundary-h or prev-boundary-h. **(B)** The *vertex move*, in which a vertex is randomly displaced. **(C)** *Single edge addition/removal.* The single edge addition move adds an edge (two half-edges) to the boundary of the shell, if the two selected boundary half-edges are bound to each other. This results in two new interactions, drawn as dark gray wedges in the schematic. The reverse move, a single edge removal, breaks two interactions and removes two half-edges form the shell. **(D)** The *paired edge addition/removal* move adds/removes two edges to the shell, resulting in forming/breaking three interactions (dark gray wedges in the schematic). **(E)** The *simple boundary bind/unbind* makes/breaks an interaction between two boundary half-edges. **(F)** *Wedge fusion bind / wedge fission unbind.* A wedge fusion move adds an interaction between two boundary half edges that are sufficiently near each other. In the example shown, in the left configuration there are two nearby boundary half-edges that can bind. This causes a vertex to be removed and adds two new interactions. The reverse move, wedge fission unbinding, results in adding a new vertex and breaking two interactions. **(G)** *Fusion bind / fission unbind* . In fusion binding, two close boundary half-edges are bound, resulting in removal of a vertex and one new interaction (dark grey wedge in the schematic). The reverse move, fission unbinding, results in removal of one vertex and breakage of one interaction. **(H)** The *conformational switch* move changes the conformations of the two half-edges within one edge. Adapted from Ref. [1], copyright 2022 ACS Publications.

bioRxiv preprint doi: https://doi.org/10.64898/2026.05.25.727729; this version posted May 26, 2026. The copyright holder for this preprint (which was not certified by peer review) is the author/funder, who has granted bioRxiv a license to display the preprint in perpetuity. It is made available under a CC-BY 4.0 International license.

vertices and edge types are unchanged (Fig S8B). The move is accepted with probability:

$$\mathrm{acc}\left(\Gamma \to \Gamma'\right) = \exp(-(\Delta\mathcal{H}_{\mathrm{elastic}} + \Delta\mathcal{H}_{\mathrm{steric}})/k_{\mathrm{B}}T), \tag{S3}$$

and the generation probability is $\mathrm{gen}\left(\Gamma \to \Gamma'\right) = 1$.

Single edge addition/removal: A half-edge $\mathbf{h}_{\mathrm{i}}$ is randomly chosen from the boundary half-edges. If $\mathbf{h}_{\mathrm{i}}$ is bound to another half-edge $\mathbf{h}_{\mathrm{j}}$ from one end, a trial is made to add a new edge, consisting of two half-edges, $\mathbf{h}_{\mathrm{k}}$ and its opposite half-edge, connecting the other end of $\mathbf{h}_{\mathrm{i}}$ to $\mathbf{h}_{\mathrm{j}}$ (Fig S8C). The generation probability is

$$\mathrm{gen}\left(\Gamma \to \Gamma'\right) = N_{\mathrm{boundary}}(\Gamma) k_{\mathrm{a}}^{0} t_0 \frac{2}{N_{\mathrm{boundary}}(\Gamma')} \tag{S4}$$

where N_{boundary} is the number of boundary half-edges, k_{a}^{0} is the trial-move rate for addition/removal moves, and t_0 is the simulation timescale, as explained in the main text.

The newly added half-edge $\mathbf{h}_{\mathrm{k}}$ interacts with $\mathbf{h}_{\mathrm{i}}$ and $\mathbf{h}_{\mathrm{j}}$, with conformation-dependent binding affinities, so the grand canonical probability of the new configuration relative to Γ is

$$\frac{P\left(\Gamma'\right)}{P(\Gamma)} = zK_{\mathrm{c(k),c(i)}} K_{\mathrm{c(j),c(k)}} \exp(-\beta\Delta\mathcal{H}) \tag{S5}$$

where $c(i), c(j)$, and $c(k)$ are the conformations of half-edges $\mathbf{h}_{\mathrm{i}}$, $\mathbf{h}_{\mathrm{j}}$, and $\mathbf{h}_{\mathrm{k}}$ respectively and $\Delta\mathcal{H} = \Delta\mathcal{H}_{\mathrm{elastic}} + \Delta\mathcal{H}_{\mathrm{steric}} + \Delta\mathcal{H}_{\mathrm{conf}}$ involves volume exclusion, the elastic energy of the new edge and its bound edges, and the conformational free energy of the new edge.

For removal of a single edge, a half-edge $\mathbf{h}_{\mathrm{r}}$ is randomly chosen from the boundary half-edges. A trial is made to remove $\mathbf{h}_{\mathrm{r}}$ and its opposite half-edge $\mathbf{h}_{\mathrm{k}}$, which includes unbinding $\mathbf{h}_{\mathrm{k}}$ from its next-h $\mathbf{h}_{\mathrm{i}}$ and prev-h $\mathbf{h}_{\mathrm{j}}$. The generation probability of edge removal is:

$$\mathrm{gen}\left(\Gamma' \to \Gamma\right) = N_{\mathrm{boundary}}\left(\Gamma'\right) k_{\mathrm{a}}^{0} t_0 \frac{1}{N_{\mathrm{boundary}}\left(\Gamma\right)} \tag{S6}$$

The acceptance criterion is:

$$\mathrm{acc}\left(\Gamma \to \Gamma'\right) = \min\left[1, \frac{zK_{\mathrm{c(k),c(i)}} K_{\mathrm{c(j),c(k)}}}{2} e^{-\beta\Delta\mathcal{H}}\right]. \tag{S7}$$

Paired edge addition/removal: In our model, edge additions that result in configurations with a dangling edge (which has only one interaction) are followed by addition of a second edge that closes the triangle. This choice is made because, under productive assembly conditions, dangling edges are highly unstable and quickly dissociate. Thus, simulations would spend the majority of their time on consecutive additions and removals of dangling edges. In previous Brownian dynamics simulations [12] we observed that in such situations net growth of assemblies was usually associated with either association of oligomers, or the rapid succession of additions of more than one subunit [12]. A similar conclusion was made from kinetic MC simulations [13]. To allow for this possibility, we include a move that enables additions and removals of dimers-of-dimers.

The two consecutive edge additions are attempted as follows: A half-edge $\mathbf{h}_{\mathrm{i}}$ is randomly chosen from the boundary half-edges. If it is open on both ends (i.e. it has neither next-h nor prev-h), a trial is made to add two new edges that bind to the selected edge. This move also includes adding a new vertex to the shell, at the intersection of the two newly added edges. To specify the coordinate of the new vertex, first the equilibrium position of the new vertex $\vec{x_0}$ is selected based on the conformations of $\mathbf{h}_{\mathrm{i}}$, $\mathbf{h}_{\mathrm{j}}$ and $\mathbf{h}_{\mathrm{k}}$. The new vertex is then displaced to $\vec{x_{\mathrm{new}}} = \vec{x_0} + \vec{\delta x}$ where the components of $\vec{\delta x}$ are selected from the Gaussian distribution $\mathcal{N}(0, \sigma_{\mathrm{th}}^2)$ with σ_{th} described above. The generation probability is

$$\mathrm{gen}\left(\Gamma \to \Gamma'\right) = N_{\mathrm{boundary}}(\Gamma) k_{\mathrm{a}}^{0} t_0 \frac{\mathcal{N}^3(0, \sigma_{\mathrm{th}}^2)}{N_{\mathrm{boundary}}(\Gamma')} \tag{S8}$$

where $\mathcal{N}^3(0, \sigma_{\mathrm{th}}^2) = \left(\mathcal{N}(0, \sigma_{\mathrm{th}}^2)\right)^3$.

The first added half-edge ($\mathbf{h}_{\mathrm{j}}$) makes a new interaction with one end of $\mathbf{h}_{\mathrm{i}}$, and the second added half-edge ($\mathbf{h}_{\mathrm{k}}$) interacts with the open ends of $\mathbf{h}_{\mathrm{j}}$ and $\mathbf{h}_{\mathrm{i}}$.

bioRxiv preprint doi: https://doi.org/10.64898/2026.05.25.727729; this version posted May 26, 2026. The copyright holder for this preprint (which was not certified by peer review) is the author/funder, who has granted bioRxiv a license to display the preprint in perpetuity. It is made available under a CC-BY 4.0 International license.

For the reverse move, a half-edge $\mathbf{h}_\mathrm{r}$ is randomly chosen from the boundary half-edges. If removal of this half-edge (and its opposite half-edge) results in a dangling edge, the dangling edge will also be removed. In this move, two edges, three interactions, and a vertex are removed. The generation probability for removal is:

$$\text{gen}\left(\Gamma' \to \Gamma\right) = N_\text{boundary}\left(\Gamma'\right) k_\mathrm{a}^0 t_0 \frac{2}{N_\text{boundary}\left(\Gamma\right)} \tag{S9}$$

and the acceptance criterion for addition is:

$$\text{acc}\left(\Gamma \to \Gamma'\right) = \min\left[1, \frac{2z^2 K_{\mathrm{c(k),c(i)}} K_{\mathrm{c(i),c(j)}} K_{\mathrm{c(j),c(k)}}}{\mathcal{N}^3(0, \sigma_\mathrm{th}^2)} e^{-\beta\Delta\mathcal{H}}\right] \tag{S10}$$

where again $\Delta\mathcal{H} = \Delta\mathcal{H}_\text{elastic} + \Delta\mathcal{H}_\text{steric} + \Delta\mathcal{H}_\text{conf}$ involves volume exclusion, the elastic energy of the new edges and their bound edges, and the conformational free energy of the new edges.

Simple boundary binding/unbinding: In a simple boundary binding, we attempt to make a new interaction between two edges whose ends are nearby but unbound to each other (Fig. S8E). For this move, a half-edge $\mathbf{h}_\mathrm{i}$ is randomly chosen from the boundary half-edges. If $\mathbf{h}_\mathrm{i}$ is open on both ends (has neither next-h nor prev-h) and makes a wedge with the next-boundary-h or prev-boundary-h $\mathbf{h}_\mathrm{j}$, (i.e., the angle between the two edges $\theta < \pi/2$) an attempt is made to bind $\mathbf{h}_\mathrm{i}$ to $\mathbf{h}_\mathrm{j}$.

For the opposite process, simple boundary unbinding, an edge is chosen randomly from the boundary edges and if it has a next-h or prev-h (it cannot have both since it is a boundary edge), an attempt is made to unbind. The acceptance criterion for simple boundary binding is:

$$\text{acc}\left(\Gamma \to \Gamma'\right) = \min\left[1, K_{\mathrm{c(i),c(j)}} e^{-\beta\Delta\mathcal{H}}\right] \tag{S11}$$

Here $\Delta\mathcal{H}$ only involves the excluded volume and elastic energies.

Wedge fusion binding/Wedge fission unbinding: For a wedge fusion move, a half-edge $\mathbf{h}_\mathrm{i}$ is randomly chosen from the boundary half-edges. A wedge fusion is attempted if: 1) $\mathbf{h}_\mathrm{i}$ makes a wedge with angle $\alpha < \pi/2$ with another boundary half-edge $\mathbf{h}_\mathrm{j}$, 2) v_in of $\mathbf{h}_\mathrm{j}$ is within δx_fuse of v_out of $\mathbf{h}_\mathrm{i}$, 3) the next-boundary-h of $\mathbf{h}_\mathrm{i}$ ($\mathbf{h}_\mathrm{k}$ in Fig S8F) is the same as prev-boundary-h of $\mathbf{h}_\mathrm{j}$, and 4) $\mathbf{h}_\mathrm{k}$ is bound to $\mathbf{h}_\mathrm{j}$ or $\mathbf{h}_\mathrm{i}$. For the implementation of this attempt, $\mathbf{v}_\mathrm{i}$ and $\mathbf{v}_\mathrm{j}$ and their associated edges are fused to a new vertex $\mathbf{v}_\mathrm{k}$ at the midpoint between $\mathbf{v}_\mathrm{i}$ and $\mathbf{v}_\mathrm{j}$. Two new interactions are made: $\mathbf{h}_\mathrm{i}$ is bound to $\mathbf{h}_\mathrm{j}$ and the third half-edge in the triangle $\mathbf{h}_\mathrm{k}$ is bound to $\mathbf{h}_\mathrm{i}$ (or to $\mathbf{h}_\mathrm{j}$).

For a wedge fission unbinding, a vertex $\mathbf{v}_\mathrm{k}$ is chosen at one end of a randomly chosen boundary half-edge. An interaction of that vertex between an associated half-edge $\mathbf{h}_\mathrm{i}$ and its next-h $\mathbf{h}_\mathrm{j}$ is randomly chosen for the wedge fission unbinding attempt. Then, v_out of the incoming half-edge $\mathbf{h}_\mathrm{i}$, with all its associated edges, is moved to the new vertex $\mathbf{v}_\mathrm{i}$ at $\vec{x}_\text{new} = \vec{x}_0 + 0.5\delta\vec{x}$; and v_in of the outgoing half-edge $\mathbf{h}_\mathrm{j}$, with all its associated edges, is moved to the new vertex $\mathbf{v}_\mathrm{j}$ at $\vec{x}_\text{new} = \vec{x}_0 - 0.5\delta\vec{x}$, where the components of $\delta\vec{x}$ are chosen from the Gaussian distribution $\mathcal{N}(0, \sigma_\mathrm{th}^2)$. To maintain the proper topology of the shell, the interaction of the third half-edge in the triangle (randomly chosen as $\mathbf{h}_\mathrm{k}$-$\mathbf{h}_\mathrm{i}$ or $\mathbf{h}_\mathrm{k}$-$\mathbf{h}_\mathrm{j}$) is also removed. The acceptance criterion for wedge fusion binding is:

$$\text{acc}\left(\Gamma \to \Gamma'\right) = \min\left[1, K_{\mathrm{c(i),c(j)}} K_{\mathrm{c(k),c(i)}} \mathcal{N}^3(0, \sigma_\mathrm{th}^2) e^{-(\beta\Delta\mathcal{H}_\text{elastic} + \Delta\mathcal{H}_\text{steric})}\right] \tag{S12}$$

Fusion binding / fission unbinding: For a fusion binding move, a half-edge $\mathbf{h}_\mathrm{i}$ is randomly chosen from the boundary half-edges. The move is attempted if: 1) $\mathbf{h}_\mathrm{i}$ forms a wedge with angle $\alpha < \pi/2$ with another boundary half-edge $\mathbf{h}_\mathrm{j}$, 2) v_in of $\mathbf{h}_\mathrm{j}$ is within δx_fuse of v_out of $\mathbf{h}_\mathrm{i}$, and 3) the next-boundary-h of $\mathbf{h}_\mathrm{i}$ ($\mathbf{h}_\mathrm{k}$ in Fig S8G) is not the same as prev-boundary of $\mathbf{h}_\mathrm{j}$. Similar to the wedge binding move, two vertices and their associated edges are fused into a new vertex $\mathbf{v}_\mathrm{k}$ at the midpoint between $\mathbf{v}_\mathrm{i}$ and $\mathbf{v}_\mathrm{j}$. This move results in an additional boundary loop in the structure (orange loop in Fig S8(G) right) and the bound vertex will be a double-boundary vertex, meaning that it is shared between two boundary loops. A boundary loop can be found by starting from a random boundary half-edge, and moving along the next-boundary-h elements until returning to the original half-edge.

A fission unbinding move is attempted if there is more than one boundary loop in the structure. A vertex $\mathbf{v}_\mathrm{k}$ is chosen at one end of a randomly chosen boundary half-edge. If this vertex is a double-boundary vertex, the fission unbinding move will be attempted, by splitting the edges ending in $\mathbf{v}_\mathrm{k}$, to form to vertices $\mathbf{v}_\mathrm{i}$ and $\mathbf{v}_\mathrm{j}$ (similar to the wedge fission unbinding move), and merging the two boundary loops. The implementation of adding the new vertex to the shell is similar to the wedge fission unbinding move, except that there is only one unbinding event ($\mathbf{h}_\mathrm{i}$-$\mathbf{h}_\mathrm{j}$).

The acceptance criterion for fusion binding is:

$$\text{acc}\left(\Gamma \to \Gamma'\right) = \min\left[1, K_{\mathrm{c(i),c(j)}} \mathcal{N}^3(0, \sigma_\mathrm{th}^2) e^{-\beta(\Delta\mathcal{H}_\text{elastic} + \Delta\mathcal{H}_\text{steric})}\right] \tag{S13}$$

bioRxiv preprint doi: https://doi.org/10.64898/2026.05.25.727729; this version posted May 26, 2026. The copyright holder for this preprint (which was not certified by peer review) is the author/funder, who has granted bioRxiv a license to display the preprint in perpetuity. It is made available under a CC-BY 4.0 International license.

Conformational switch: A half-edge $\mathbf{h}_\mathrm{i}$ is randomly chosen from the set of all edges in the structure, and a trial is made to change the conformation of $\mathbf{h}_\mathrm{i}$ from $c(i)$ to $c'(i)$ and the conformation of its opposite half-edge $\mathbf{h}_\mathrm{j}$ from $c(j)$ to $c'(j)$. The generation probability is $\mathrm{gen}\left(\Gamma \to \Gamma'\right) = 1$, and the move is accepted with probability:

$$\mathrm{acc}\left(\Gamma \to \Gamma'\right) = \min\left[1, \frac{K_{c'(i),c(p)}K_{c(q),c'(i)}K_{c'(j),c(r)}K_{c(s),c'(j)}}{K_{c(i),c(p)}K_{c(q),c(i)}K_{c(j),c(r)}K_{c(s),c(j)}} e^{-\beta(\Delta\mathcal{H}_{\mathrm{elastic}}+\Delta\mathcal{H}_{\mathrm{conf}})}\right], \tag{S14}$$

where p and q are the indices of the next-h and prev-h of i, and r and s are the indices of the next-h and prev-h of j.

CAM binding/unbinding: For a CAM binding move, a half-edge $\mathbf{h}_\mathrm{i}$ is randomly chosen. If the half-edge is type CD or DC and has a prev-h, and no CAM is bound already, then an attempt is made to add a CAM to the interface between $\mathbf{h}_\mathrm{i}$ and prev-h (for the purposes of bookkeeping, we associate the CAM with $\mathbf{h}_\mathrm{i}$ rather than prev-h; see Fig. S9A). The generation probability is:

$$\mathrm{gen}\left(\Gamma' \to \Gamma\right) = k^0_{\mathrm{CAM}} t_0 \tag{S15}$$

The move is accepted with probability:

$$\mathrm{acc}\left(\Gamma \to \Gamma'\right) = \min\left[1, K_{\mathrm{CAM},c,c'} z_{\mathrm{CAM}} e^{-\beta\Delta\mathcal{H}}\right]. \tag{S16}$$

Similarly, for a CAM unbinding move, a half-edge is randomly chosen. If the half-edge is type CD or DC and a CAM is bound to its interface with prev-h (if prev-h exists), then an attempt is made to remove the CAM from the interface. The generation probability for such moves is:

$$\mathrm{gen}\left(\Gamma' \to \Gamma\right) = k^0_{\mathrm{CAM}} t_0, \tag{S17}$$

and the acceptance probability is:

$$\mathrm{acc}\left(\Gamma' \to \Gamma\right) = \min\left[1, \left(K_{\mathrm{CAM},c,c'} z_{\mathrm{CAM}}\right)^{-1} e^{\beta\Delta\mathcal{H}}\right]. \tag{S18}$$

In principle, the procedure detailed above could result in CAMs being bound to the interface between CD/DC and AB/BA dimers. However, because we set $g^{\mathrm{CAM}} = 0$ for any interfaces that are not C-D or D-C, in practice CAM is only ever observed bound to C-D and D-C interfaces.

CAM-bound dimer-of-dimers addition/removal: We also allow for the binding and unbinding of CAM-bound CD dimers-of-dimers (Fig. S9B). The rules are the same as for paired edge addition/removal; one simply accounts for the additional energy of the CAM bound to the dimer-of-dimers interface. Importantly, dimers-of-dimers cannot be removed if CAMs are bound to either of the other dimer-of-dimers interfaces in the triangle to which the selected half-edge belongs (see Fig. S9C).

bioRxiv preprint doi: https://doi.org/10.64898/2026.05.25.727729; this version posted May 26, 2026. The copyright holder for this preprint (which was not certified by peer review) is the author/funder, who has granted bioRxiv a license to display the preprint in perpetuity. It is made available under a CC-BY 4.0 International license.

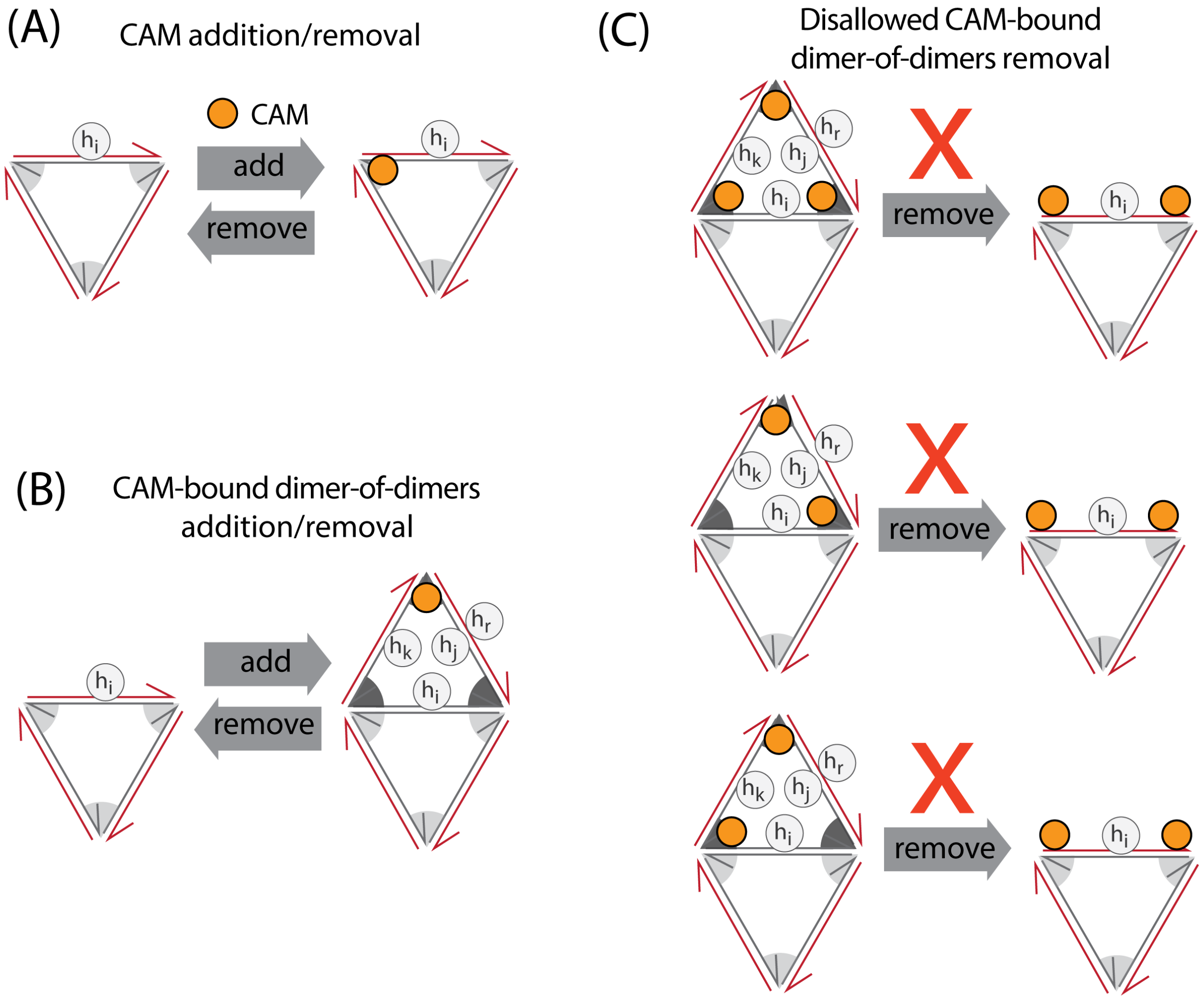

FIG. S9. **Schematic of MC moves involving CAMs.** **(A)** The *CAM addition/removal* move adds/removes a CAM to/from the interface between two bound edges. **(B)** The *CAM-bound dimer-of-dimers addition/removal* move adds/removes two edges with a CAM bound to their interface to the shell, resulting in forming/breaking three interactions (dark gray wedges in the schematic). **(C)** CAM-bound dimer-of-dimers removal moves that would result in CAMs on boundary half-edges are disallowed.

bioRxiv preprint doi: https://doi.org/10.64898/2026.05.25.727729; this version posted May 26, 2026. The copyright holder for this preprint (which was not certified by peer review) is the author/funder, who has granted bioRxiv a license to display the preprint in perpetuity. It is made available under a CC-BY 4.0 International license.

Ionic strength (mM)	$g^{T=4}_{\text{per-dimer}}(k_\text{B}T)$	$g^{\text{sheet}}_{\text{per-dimer}}(k_\text{B}T)$	$g^{T=4,\text{ w/ CAM}}_{\text{per-dimer}}(k_\text{B}T)$	$g^{\text{sheet, w/ CAM}}_{\text{per-dimer}}(k_\text{B}T)$
1000	-0.90	3.70	-2.35	-1.64
300	-0.50	3.64	-1.95	-1.71
80	-0.10	3.57	-1.55	-1.80

TABLE S3. Values of per-subunit binding free energies, with and without CAMs, at different ionic strengths.

S3. FREE ENERGIES OF CAPSIDS AND SHEETS

The per-dimer free energy of $T = 4$ capsids and sheets depends upon both ionic strength and the presence of CAMs. Without CAMs, the per-dimer free energies of $T = 4$ capsids ($g^{T=4}_{\text{per-dimer}}$) and sheets ($g^{\text{sheet}}_{\text{per-dimer}}$) are given by:

$$g^{T=4}_{\text{per-dimer}} = 2(g^{\text{bind}}_{T=4} + g_{\text{ent}}) - \mu_{\text{dimer}} \tag{S19}$$

$$g^{\text{sheet}}_{\text{per-dimer}} = 2\left((g_{\text{C-D}} + g_{\text{D-C}})/2 + g_{\text{ent}}\right) - \mu_{\text{dimer}}, \tag{S20}$$

where $g_{\text{ent}} \approx 3.42 k_\text{B}T$ [1] is the entropic penalty for dimer-dimer binding and the factors of two account for the fact that each dimer in a capsid is bound to four other dimers. The expression for $g^{\text{sheet}}_{\text{per-dimer}}$ neglects the cost associated with the boundary of the sheet. Alternatively, if CAMs are bound to each possible site in the structures, the per-subunit free energies are:

$$g^{T=4,\text{ w/ CAM}}_{\text{per-dimer}} = 2(g^{\text{bind}}_{T=4} + g_{\text{ent}}) - \mu_{\text{dimer}} + g^{\text{CAM}}_{\text{C-D}}/2 - \mu_{\text{CAM}}/2 \tag{S21}$$

$$g^{\text{sheet, w/ CAM}}_{\text{per-dimer}} = 2\left((g_{\text{C-D}} + g_{\text{D-C}})/2 + g_{\text{ent}}\right) - \mu_{\text{dimer}} + g^{\text{CAM}}_{\text{C-D}}/2 + g^{\text{CAM}}_{\text{D-C}}/2 - \mu_{\text{CAM}}. \tag{S22}$$

Values of $g^{T=4}_{\text{per-dimer}}$, $g^{\text{sheet}}_{\text{per-dimer}}$, $g^{T=4,\text{ w/ CAM}}_{\text{per-dimer}}$, and $g^{\text{sheet, w/ CAM}}_{\text{per-dimer}}$ for different ionic strengths are shown in Table S3.

bioRxiv preprint doi: https://doi.org/10.64898/2026.05.25.727729; this version posted May 26, 2026. The copyright holder for this preprint (which was not certified by peer review) is the author/funder, who has granted bioRxiv a license to display the preprint in perpetuity. It is made available under a CC-BY 4.0 International license.

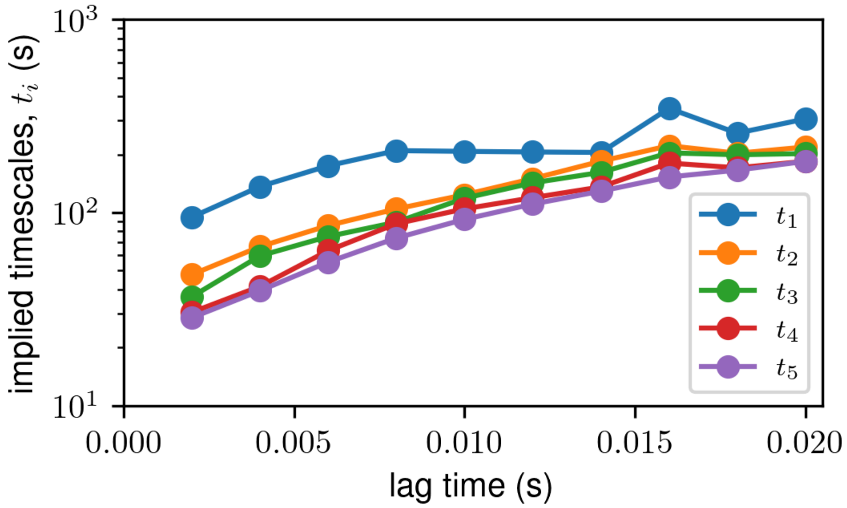

FIG. S10. **Implied timescales vary slowly with lag time.** Plot showing implied timescales $t_i = -\tau/\log \lambda_i$ computed by diagonalizing the MSM transition matrix versus the lag time (τ) in seconds. We show the five longest timescales in different colors, corresponding to the five highest eigenvalues λ_i of the transition matrix with $\lambda_i < 1$.

S4. MARKOV STATE MODEL VALIDATION

To validate our Markov state model (MSM), we perform two tests. In the first test (widely used in the MSM literature, e.g. [14]), we compute implied timescales of the MSM for varying lag times τ. Implied timescales t_i are defined in terms of the eigenvalues λ_i of the MSM transition matrix $\boldsymbol{T}(\tau)$:

$$t_i = -\tau/\log(\lambda_i). \tag{S23}$$

If the dynamics are Markovian, then t_i should be independent of τ. In practice this is almost never exactly true, and instead a rule of thumb is that t_i should vary only slowly with τ for the largest implied timescales (i.e. largest eigenvalues with $\lambda_i < 1$). Fig. S10 shows that this is indeed the case for the largest five implied timescales in our MSM.

As a second test, we compute the yields of $T = 4$ capsids and sheets as a function of time with the MSM, and compare them to yields computed directly from KMC simulations. This is a much more stringent test of the MSM than the implied timescale test, because it requires that all elements of the transition matrix be accurately estimated. The MSM yield versus time is given by $\sum_{i\in B} p_i(t)$, where the sum is over microstates i associated with the product state B ($T = 4$ capsid or sheet) and $p_i(t)$ is computed via Eq. 11 in the main text. The KMC yield is the fraction of trajectories in which a product ($T = 4$ capsid or sheet) has formed. Fig. S11 shows that the MSM yield dynamics agrees very well with that of the brute-force KMC simulations.

bioRxiv preprint doi: https://doi.org/10.64898/2026.05.25.727729; this version posted May 26, 2026. The copyright holder for this preprint (which was not certified by peer review) is the author/funder, who has granted bioRxiv a license to display the preprint in perpetuity. It is made available under a CC-BY 4.0 International license.

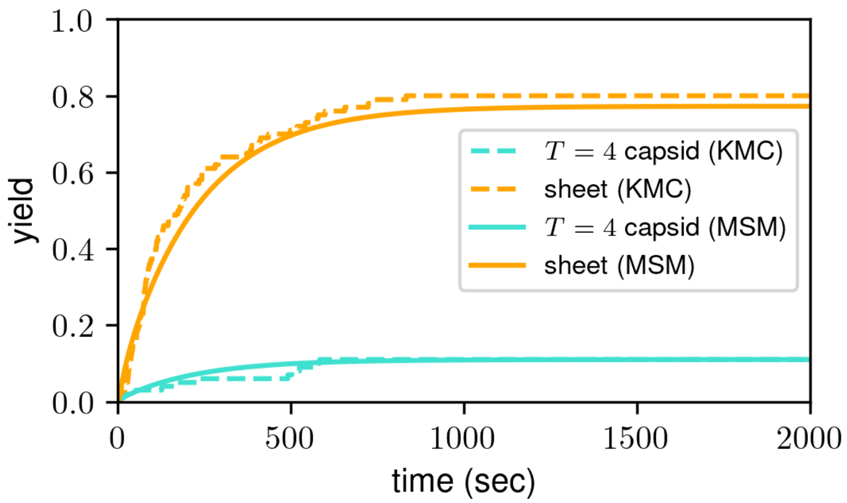

FIG. S11. **MSM dynamics agree well with KMC dynamics.** Yield, i.e. the fraction of trajectories ending in either $T = 4$ capsids (turquoise) or sheets (orange), versus time. The dashed lines show results from KMC simulations, while the solid lines show results from the MSM (obtained by solving Eq. 11 in the main text).

bioRxiv preprint doi: https://doi.org/10.64898/2026.05.25.727729; this version posted May 26, 2026. The copyright holder for this preprint (which was not certified by peer review) is the author/funder, who has granted bioRxiv a license to display the preprint in perpetuity. It is made available under a CC-BY 4.0 International license.

S5. MOVIE DESCRIPTIONS

- **Movie S1**: Trajectory showing $T = 4$ capsid assembly with CAMs at $I = 1000$mM. The corresponding assembly size versus time plot is shown in the main text (Fig. 3A).
- **Movie S2**: Trajectory showing malformed assembly with mixed $T = 3/T = 4$ morphology with CAMs at $I = 1000$mM. The corresponding assembly size versus time plot is shown in the main text (Fig. 3B).
- **Movie S3**: Trajectory showing $T = 4$ capsid assembly with CAMs at $I = 300$mM. The corresponding assembly size versus time plot is shown in main text (Fig. 3C).
- **Movie S4**: Trajectory showing malformed assembly with mixed sheet/$T = 4$ morphology with CAMs at $I = 300$mM. The corresponding assembly size versus time plot is shown in the main text (Fig. 3D).
- **Movie S5**: Trajectory showing sheet assembly with CAMs at $I = 80$mM. The corresponding assembly size versus time plot is shown in the main text (Fig. 3F).
- **Movie S6**: Trajectory showing $T = 4$ capsid assembly without CAMs at $I = 300$mM.

[1] F. Mohajerani, B. Tyukodi, C. J. Schlicksup, J. A. Hadden-Perilla, A. Zlotnick, and M. F. Hagan, Multiscale Modeling of Hepatitis B Virus Capsid Assembly and Its Dimorphism, ACS Nano **16**, 13845 (2022).

[2] L. Kettner, Designing a data structure for polyhedral surfaces, in *Proceedings of the Fourteenth Annual Symposium on Computational Geometry - SCG '98* (ACM Press, Minneapolis, Minnesota, United States, 1998) pp. 146–154.

[3] D. E. Muller and F. P. Preparata, Finding the intersection of two convex polyhedra, Theoretical Computer Science **7**, 217 (1978).

[4] S. Li, R. Zandi, A. Travesset, and G. M. Grason, Ground States of Crystalline Caps: Generalized Jellium on Curved Space, Physical Review Letters **123**, 145501 (2019).

[5] G. M. Rotskoff and P. L. Geissler, Robust nonequilibrium pathways to microcompartment assembly, Proceedings of the National Academy of Sciences **115**, 6341 (2018).

[6] W. Wu, N. R. Watts, N. Cheng, R. Huang, A. C. Steven, and P. T. Wingfield, Expression of quasi-equivalence and capsid dimorphism in the Hepadnaviridae, PLOS Computational Biology **16**, e1007782 (2020).

[7] R. Asor, L. Selzer, C. J. Schlicksup, Z. Zhao, A. Zlotnick, and U. Raviv, Assembly Reactions of Hepatitis B Capsid Protein into Capsid Nanoparticles Follow a Narrow Path through a Complex Reaction Landscape, ACS Nano **13**, 7610 (2019).

[8] P. Buzón, S. Maity, P. Christodoulis, M. J. Wiertsema, S. Dunkelbarger, C. Kim, G. J. Wuite, A. Zlotnick, and W. H. Roos, Virus self-assembly proceeds through contact-rich energy minima, Science Advances **7**, eabg0811 (2021).

[9] R. C. Oliver, W. Potrzebowski, S. M. Najibi, M. N. Pedersen, L. Arleth, N. Mahmoudi, and I. André, Assembly of Capsids from Hepatitis B Virus Core Protein Progresses through Highly Populated Intermediates in the Presence and Absence of RNA, ACS Nano **14**, 10226 (2020).

[10] N. Metropolis, A. W. Rosenbluth, M. N. Rosenbluth, A. H. Teller, and E. Teller, Equation of State Calculations by Fast Computing Machines, The Journal of Chemical Physics **21**, 1087 (1953).

[11] W. K. Hastings, Monte Carlo sampling methods using Markov chains and their applications, Biometrika **57**, 97 (1970).

[12] M. F. Hagan and D. Chandler, Dynamic Pathways for Viral Capsid Assembly, Biophysical Journal **91**, 42 (2006).

[13] T. Zhang and R. Schwartz, Simulation Study of the Contribution of Oligomer/Oligomer Binding to Capsid Assembly Kinetics, Biophysical Journal **90**, 57 (2006).

[14] W. C. Swope, J. W. Pitera, F. Suits, M. Pitman, M. Eleftheriou, B. G. Fitch, R. S. Germain, A. Rayshubski, T. J. C. Ward, Y. Zhestkov, and R. Zhou, Describing Protein Folding Kinetics by Molecular Dynamics Simulations. 2. Example Applications to Alanine Dipeptide and a β-Hairpin Peptide, The Journal of Physical Chemistry B **108**, 6582 (2004).
